# The value of necropsy reports for animal health surveillance

**DOI:** 10.1186/s12917-018-1505-1

**Published:** 2018-06-18

**Authors:** Susanne Küker, Celine Faverjon, Lenz Furrer, John Berezowski, Horst Posthaus, Fabio Rinaldi, Flavie Vial

**Affiliations:** 1 Veterinary Public Health Institute, Vetsuisse Faculty, University of Bern, Schwarzenburgstrasse 155, 3097, Liebefeld, Switzerland; 2 Institute of Computational Linguistics, University of Zürich, Andreasstrasse 15, 8050 Zürich, Switzerland; 3 Institute of Animal Pathology, University of Bern, Länggassstrasse 122, 3012 Bern, Switzerland; 4 Present Address: Epi-Connect, Skogås, Sweden

**Keywords:** Electronic necropsy records, Veterinary, Informatics, Surveillance, Text-mining

## Abstract

**Background:**

Animal health data recorded in free text, such as in necropsy reports, can have valuable information for national surveillance systems. However, these data are rarely utilized because the text format requires labor-intensive classification of records before they can be analyzed with using statistical or other software. In a previous study, we designed a text-mining tool to extract data from text in necropsy reports. In the current study, we used the tool to extract data from the reports from pig and cattle necropsies performed between 2000 and 2011 at the Institute of Animal Pathology (ITPA), University of Bern, Switzerland. We evaluated data quality in terms of credibility, completeness and representativeness of the Swiss pig and cattle populations.

**Results:**

Data was easily extracted from necropsy reports. Data quality in terms of completeness and validity varied a lot depending on the type of data reported. Diseases of the gastrointestinal system were reported most frequently (54.6% of pig submissions and 40.8% of cattle submissions). Diseases affecting serous membranes were reported in 16.0% of necropsied pigs and 27.6% of cattle. Respiratory diseases were reported in 18.3% of pigs and 21.6% of cattle submissions.

**Conclusions:**

This study suggests that extracting data from necropsy reports can provide information of value for animal health surveillance. This data has potential value for monitoring endemic disease syndromes in different age and production groups, or for early detection of emerging or re-emerging diseases. The study identified data entry and other errors that could be corrected to improve the quality and validity of the data. Submissions to veterinary diagnostic laboratories have selection biases and these should be considered when designing surveillance systems that include necropsy reports.

**Electronic supplementary material:**

The online version of this article (10.1186/s12917-018-1505-1) contains supplementary material, which is available to authorized users.

## Background

In veterinary public health, active and passive surveillance most often relies on the definitive diagnosis of specific diseases using laboratory tests for known pathogens. However, when a previously unknown or unexpected pathogen emerges in a population, this approach can take additional time and many result in a delay in pathogen identification and detection [1–3]. The same may apply to multifactorial diseases such as production diseases where detection of a single pathogen is often insufficient to unravel the underlying causal factors contributing to the disease problem.

Syndromic surveillance systems (SYS) were developed to enhance traditional passive surveillance systems and for this reason they are currently increasing in importance in veterinary medicine [4–6]. Through the analysis and interpretation of pre-diagnostic health-related data, veterinary SYS supports the early identification of disease threats that may require veterinary public health action [7]. Various sources of data have been evaluated for use in veterinary SYS worldwide [6]. These include clinical observations collected from veterinary practitioners [8], diagnostic laboratory test requests [9], post-mortem meat inspection information [10–14] and examinations of fallen stock [15–18]. Data quality in terms of completeness, validity, representativeness, usefulness and timeliness are important characteristics for determining the effectiveness of a SYS for detecting changes in animal health [19].

Necropsies performed by diagnostic laboratories on fallen stock or diseased animals could provide additional information about animal health that could have value for SYS [4]. Necropsies are a common diagnostic tool in veterinary medicine and play an important role in the investigation of disease outbreaks [20–22]. Veterinarians may be called by farmers to investigate a sudden increase in morbidity or mortality of an unknown or unrecognized disease. If animals died suspiciously or were euthanized, veterinarians may perform a field necropsy or arrange for the transport of the carcasses to a diagnostic laboratory, where a more detailed post-mortem examination and diagnostic workup can take place [23, 24]. In most disease investigations involving food-producing animals, the main purpose of a post-mortem examination is to provide additional information about the cause of death. The information present in necropsy reports is in the form of patho-morphological diagnoses, which are very specific descriptions of the gross and microscopic lesions, and the results of diagnostic tests. They also contain information about the geographic location of the farm, the affected animal(s), species, breed, sex, age, production group, clinical presentation of affected animal(s), and other farm-level characteristics, which are valuable for disease surveillance [4].

Necropsy reports could potentially have value for identifying unexpected health events that occur in a population. Syndromes representing a broad range of diseases could be defined and monitored in a SYS based on pathological findings from necropsies. Recent examples of the use of syndromes from pathology data for disease surveillance include abortion and fetal anomalies that occurred in the Schmallenberg virus epidemic in Europe [25] and central nervous system lesions in cattle infected with Astroviruses [26]. These examples suggest that monitoring of necropsy data from food production animals can be of value for early detection of new diseases or detection of changing endemic disease patterns in a population [21]. However, necropsy data from food production animals are rarely used for SYS. One challenge for using necropsy reports in routine surveillance is that these reports are commonly created and stored in natural language (free text format). This makes it difficult to extract the structured data required for the computer based data manipulation and analysis needed for surveillance. Furthermore, no internationally accepted unifying clinical terminology or coding system, such as the International Classification of Diseases (ICD) developed for human medicine, exists in animal health, although there have been attempts to create such systems [27, 28]. This makes automated classification and integration of multiple datasets difficult [4, 27].

Natural language processing (NLP) methods such as text mining are becoming increasingly important for dealing with natural language records [29]. Text mining is used to extract data from text found in electronic documents such as medical records, by automatically classifying text data into a defined set of categories which are monitored over time [30]. This approach has been used in human [31, 32] and in animal health surveillance [33–35]. However, text mining has not yet been systematically applied to livestock necropsy reports.

The objective of our study was to evaluate the quality of the data extracted from necropsy reports from food animals in order to estimate their value for SYS. A text-mining tool that classifies necropsy reports based on topographical organ systems was developed in a previous study [36] and used in the current study to classify necropsy reports into syndromes that could be used for SYS. We used these results to estimate the distribution of syndromes in the population and make inferences about the applicability of the tool and necropsy reports for veterinary SYS.

## Methods

### Necropsy data

We analyzed 12 years of pig and cattle necropsy data recorded at the Institute of Animal Pathology (ITPA), Vetsuisse Faculty, University of Bern, Switzerland. Post-mortem findings were entered manually as free text by veterinary pathologists, using a record keeping and reporting software application and database (Qualicare, Qualidoc AG). Final necropsy reports contained several separate sections, such as macroscopic findings, histologic findings and morphological diagnoses, as well as results from additional diagnostic tests such as microbiological investigations.

Necropsy reports were extracted from the Qualicare software as a Microsoft Excel file. The dataset (*n* = 6031 pig records and *n* = 2911 cattle records) included necropsies performed on whole carcasses. Reports from examinations of partial carcasses, entries without text and double entries were excluded.

Necropsy reports included the following additional variables: a unique necropsy submission number, species, breed, sex, weight, date, animal age (in days), how the animal died (e.g., euthanized) and address of the owner. For cattle, the breed and the unique animal identification number registered in the Swiss central animal movement database (Tierverkehrsdatenbank, TVD) were also recorded. Information about the clinical history of necropsied animals was not available in 99% of the reports and for this reason clinical history was excluded from study.

### Data quality

Data quality was assessed by evaluating the completeness, validity, and representativeness of the data [19]. Data quality was assessed for the following fields: Submission number, Sex, Age (in days), Weight (in Kg), How the animal died, Owner name, Owner address, (postal code) Breed, TVD number. Complete records were records that had an entry in the record for the field that was valid. Entries were valid if they were logically or biologically plausible.

Age and weight validity were assessed by checking for biologically unlikely entries (e.g., > 250 kg weight for pigs were classified as invalid). For cattle reports, the unique animal identification number (TVD number) was classified as invalid if it was not exactly 8 digits long or contained punctuation or letters.

The number of dead cattle and pigs was not available for the full period of the study. Therefore, to assess the representativeness of the submissions, the distributions of sex, age, breed and geographical origin of necropsied animals were compared to those of the overall Swiss pig and cattle living populations using data provided by the Swiss Federal Veterinary Office.

### Syndrome categories & text-mining classification tool

We defined 12 syndrome categories based primarily on affected organ systems. This classification included the following systems: gastrointestinal (GI), respiratory (RESP), heart (HEART), lymphatic (LYMPH), nervous (NEURO), musculoskeletal (MUSCO), urinary (URI), reproductive (REPRO) and serous membrane systems (SERO). Three additional non-organ system syndrome categories were defined, one for congenital malformations (MAL), one for neoplasia (NEO) and one named OTHER for diagnoses or terms that referred to pathologic patterns that could not be assigned to one of the previous syndrome categories. Examples of reports classified as OTHER included intoxications, macroscopic findings of the skin and septicemia (details can be found in Additional file 1). We constructed a German veterinary terminology resource to classify necropsy reports into at least one of the above syndrome categories. Each report could be classified into more than one category. The construction of the ontology is thoroughly described in [36] but is summarized below. We started with a frequency-ranked list of the words found in all of the reports and implemented methods for dealing with spelling mistakes, inflections, negations and abbreviations. We selected “key terms” that were likely to indicate diagnoses in the reports. These key terms (*n* = 484) were mostly morphological diagnoses (e.g., bronchopneumonia or enteritis), macroscopic findings (e.g., “bladder stone”, which relates to urolithiasis), diagnosis synonyms (e.g., “Sohlengeschwür-pododermatitis”) or sub terms (e.g., “jejunitis”, which can be stated as enteritis). The key terms were then assigned to the syndrome categories listed above (Table 1 and Additional file 1). A further selection criterion was the proportion of correctly classified reports: if a key term resulted in many incorrect classifications, it was excluded. The evaluation of classification is reported in [36].

**Table 1:** List of the 12 syndrome categories used for classification of necropsy records by a text-mining tool, with examples of key terms that resulted in classification into the categories

Syndromic category	Examples of diagnoses
Gastrointestinal system	enteritis, colon perforation, mesenteric torsion, typhlocolitis, abomasitis
Respiratory system	pneumonia, bronchitis, laryngitis, sinusitis, tracheitis
Heart	endocarditis, cardiomyopathy, heart infarct, myocarditis
Lymphatic system	lymphadenitis, splenitis, lymphomegaly, tonsillitis, splenic torsion
Reproductive system	abortion, ovarian cyst, metritis, uterus torsion, vaginitis
Urinary system	cystitis, kidney cyst, tubular necrosis, nephritis, bladder rupture
Neurologic system	encephalitis, meningitis, brain edema, paralysis, brain abscess
Musculoskeletal system	arthrosis, callus, fracture, muscle degeneration, osteochondrosis
Other	hydrothorax, intoxication, otitis, skin perforation, dermatitis
Congenital malformation	atresia, ectopy, heart malformation, septal defect, malformation
Neoplasia	osteosarcoma, tumor, neoplasia, carcinoma, metastases
Serous membranes	peritonitis, pleuritis, pericarditis, polyserositis, serositis

After classification by the text-mining tool, we analyzed the most common syndrome categories for each species, sex and age. Special attention was paid to records that were not classified. Unclassified records either did not contain any pathological information or they contained words that were not used for classification because they would have increased the number of misclassifications.

### Statistical analysis

Descriptive analyses were performed using R version 3.2.4 [37] with the packages: ggplot2 [38], rgdal [39], rgeos [40] and RColorBrewer [41]. The shape file used to plot the maps of Switzerland was obtained from the Swiss cadastral system (https://www.swisstopo.admin.ch/en/knowledge-facts/swiss-cadastral-surveying.html).

Time series analysis was conducted on the total number of submissions for each species and on the three most frequent syndromes per species. Three temporal patterns were investigated: long-term trend, monthly effect and day-of-the-week effect. Trend was defined as a continuous time variable. Months and days of the week were included using categorical variables for each month and day. A zero-inflated negative binomial model was fitted to the daily time-series to evaluate the effect of day of the week using the R package *pscl* [42]. A negative binomial model was used to evaluate the trend and the monthly effect with the *MASS* R package [43]. Models including temporal patterns were compared with the null model using likelihood ratio tests with a statistical significance level of 0.05.

The pig age classification of our dataset was linked to that of the Swiss pig industry, in which the age is classified based on the production status of the pig, as follows: piglets (1 to 4 weeks), weaner pigs (> 4 to 10 weeks), fattening pigs (> 10 to 22 weeks) and adults (> 22 weeks). Cattle were grouped into the following age classes: 0 to 1 month, > 1 to 6 months, > 6 months to 1 year, > 1 to 2 years and > 2 years.

## Results

### Data quality

The variables date, owner name and submission number were the most complete with 0 to 0.3% incomplete or invalid entries (Table 2). The variables: age, breed and production type (the latter two being cattle-specific) had 20 to 35% missing or invalid entries. The TVD number was missing or improperly transcribed in 80% of the cattle reports. A small number of zip codes of owner addresses were missing in the dataset, and 4.5% for pigs and 3.5% for cattle were invalid. Only a small proportion of necropsied animals had no geographical information associated with them (1.2% of pigs and 2% of cattle).

**Table 2:** Data quality (completeness and validity) for the 13 descriptive variables extracted from the post-mortem reports for pigs and cattle between 2000 and 2011. The TVD number is the unique Swiss cattle identification number

	Cattle			Pig			Cattle	Pig
	Complete	Missing	Invalid	Complete	Missing	Invalid	% unusable entries	% unusable entries
Submission number	2862	0	0	5997	0	0	0	0
Sex	2753	109	0	5823	174	0	4	3
Age (days)	2114	549	199	4361	1471	165	27	27
Weight (kg)	2742	1	119	5888	1	108	4	2
How animal died	2786	76	0	5901	96	0	3	2
Owner name	2862	0	0	5996	1	0	0	< 1
Owner address	2611	251	0	5809	188	0	9	3
Owner address (zip code)	2847	15	0	5983	14	0	< 1	< 1
Breed	2279	583	0	NA	NA	NA	21	NA
TVD number	1604	9	1284	NA	NA	NA	80	NA

### Descriptive analysis of necropsied animals

**Fig. 1:**
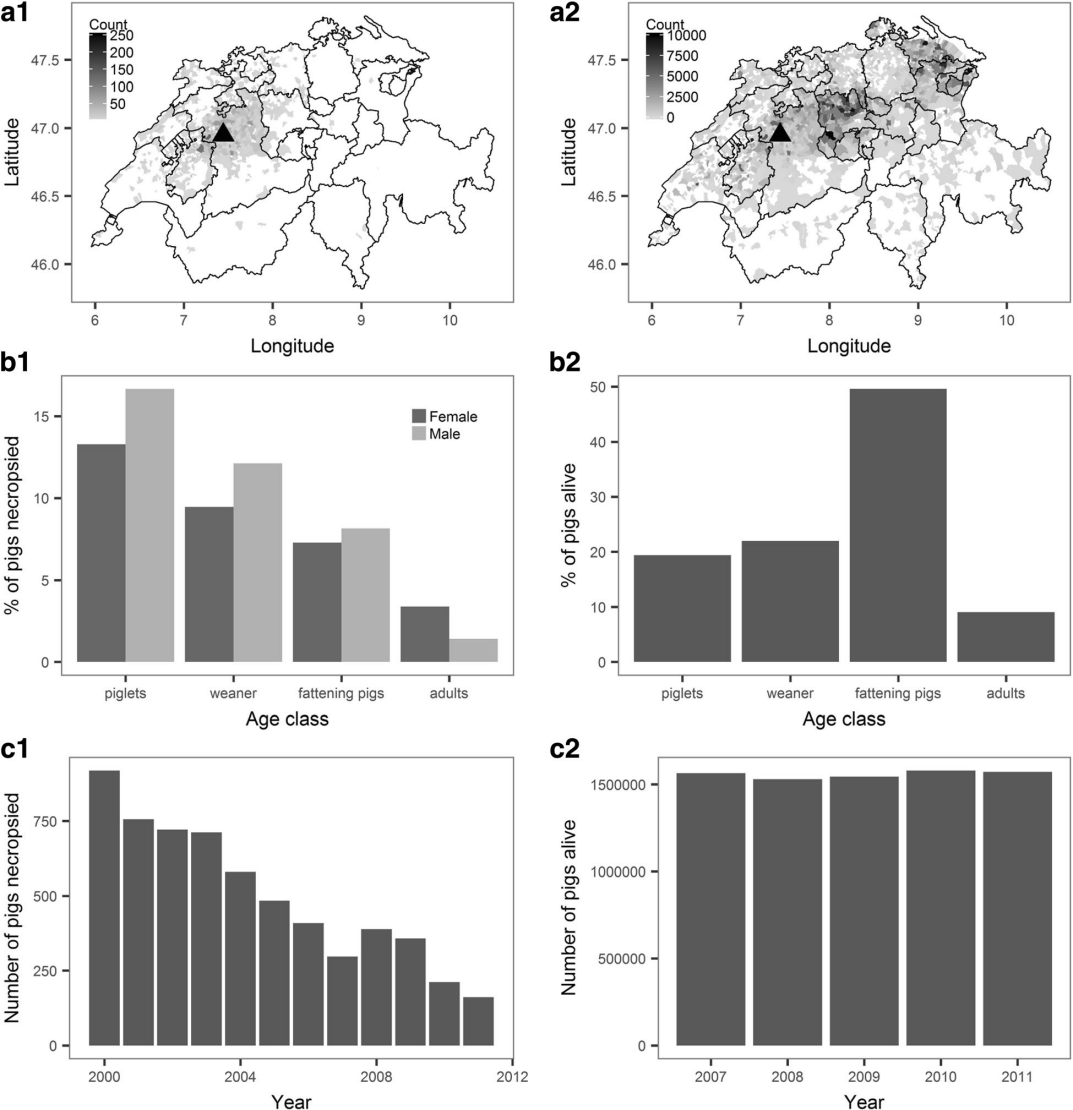
Results for pigs. The left row (1) presents results of data collected at the Animal Pathology Institute (ITPA) of the University of Bern, Switzerland in 2000–2012. The right row (2) presents data for the pig population in 2007–2011 in Switzerland, provided by the farm census database (Agrarpolitisches Information system AGIS, Federal Office for Agriculture FOAG). Data were only available for the period 2007–2011; the sex ratio could not be determined. Column A represents the geographical distribution of pigs (“count” = number of pigs); column B the age groups and their ex ratio and column C the number of animals per year

**Fig. 2:**
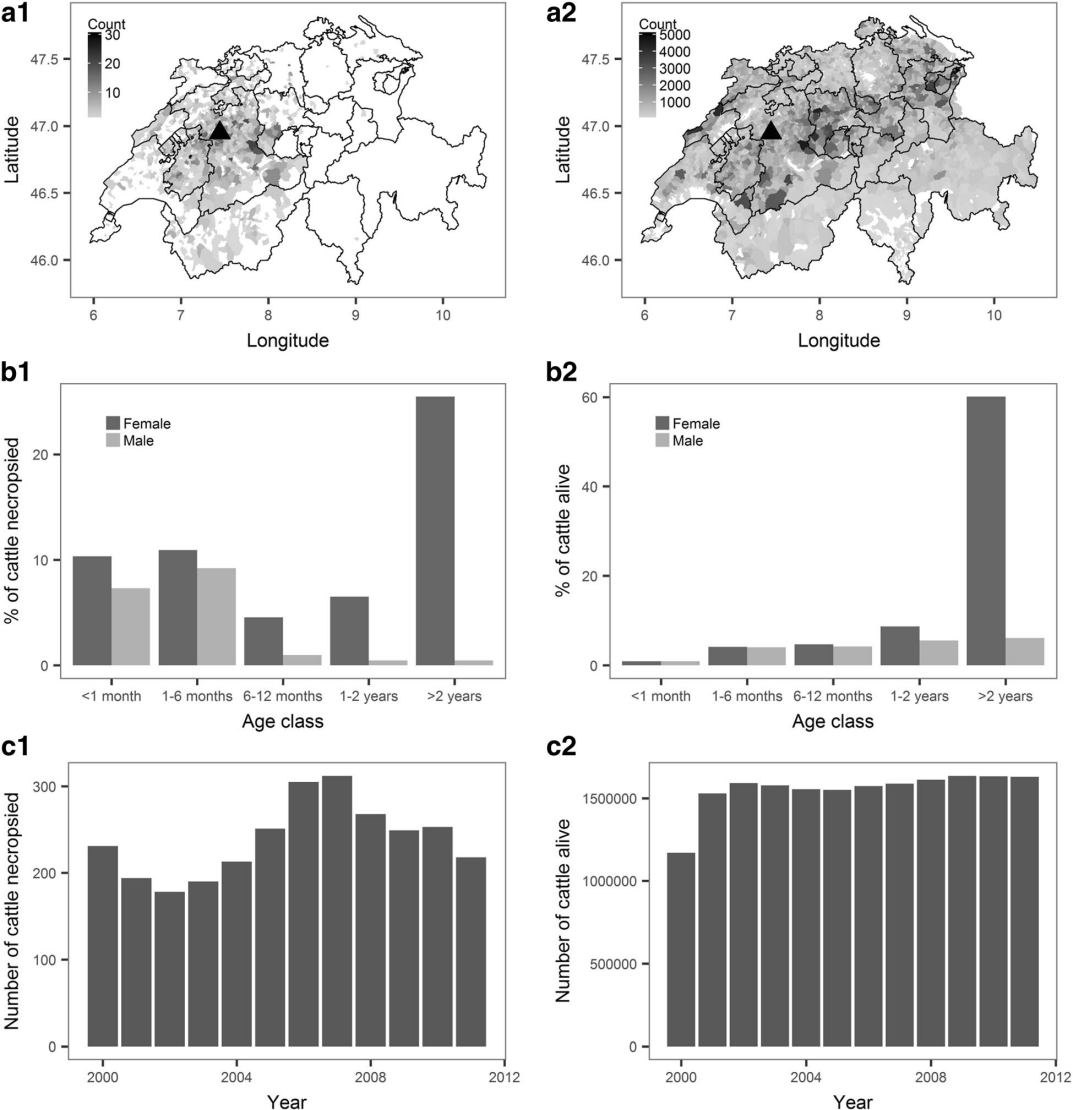
Results for cattle. The left row (1) presents results of data collected at the Animal Pathology Institute (ITPA) of the University of Bern, Switzerland in 2000–2012. The right row (2) presents data for the cattle population in 2000–2011 in Switzerland, provided by the Swiss central animal movement database (TVD database 2014; http://www.tierverkehr.ch). Column A represents the geographical distribution of cattle (“count” = number of cattle), column B the age groups and their ex ratio and column C the number of animals per year

Necropsied animals submitted to the ITPA originated mostly from west and central parts of Switzerland (Fig. 1.a1 for pigs and Fig. 2.a1 for cattle; “count” = number of animals). Cattle submissions came from a larger area of Switzerland than swine submissions. Registered pig and cattle farms were more widely distributed throughout Switzerland (Fig. 1.a2 for pigs and Fig. 2.a2 for cattle, “count” = number of animals); indicating that submissions to the ITPA did not provide complete coverage of the Swiss cattle or and swine populations.

The proportion of males to female pigs was slightly higher (2258 males to 1834 females). The majority of pigs were piglets (1–4 weeks of age) or weaner (> 4–10 weeks of age), (Fig. 1.b1). This contrasts with the age distribution of the pig population in Switzerland, where the majority of pigs are fattening pigs (11–22 weeks of age; Fig. 1.b2).

The greatest proportion of necropsied cattle (26%) were in the age class “2 years and above” (Fig. 2.b1). These animals were almost exclusively (702/715) females. However, when the < 1 month and 1–6 month categories were combined, we found that 53% of necropsied cattle (586/1040 females) were 6 months of age or younger. This age distribution is in contrast to the Swiss cattle population, which consists predominately of female cattle over 2 years old (Fig. 2.b2).

The most common breeds of necropsied cattle were Holstein Friesian (*n* = 573), Simmental Fleckvieh (*n* = 371), Red Holstein (*n* = 365), and crosses of Simmental Fleckvieh and Red Holstein (*n* = 250), which aligns with the main breeds reported to be present in the Swiss cattle population [44]. However, data on the breed distribution of cattle in Switzerland were not available to make direct comparisons.

### Syndrome classification

The four most frequent syndrome categories were the same for both species: GI (pigs 54.6%, cattle 40.8%), SERO (pigs 16.0%, cattle 27.6%), RESP (pigs 18.3%, cattle 21.6%) and OTHER (pigs 14.7%, cattle 13.8%). In cattle, REPRO also represented a large proportion of necropsies (19.1%), but this syndrome was less frequently encountered in pigs (4.4%). Thirty-six percent of pig necropsy reports and 45% of cattle necropsy reports were classified into more than one syndrome. The most frequent combinations of syndromes were GI and/or RESP and/or SERO and/or OTHER for both species. The majority (90.2%) of post-mortem records were classified into at least one category. The main reason reports were not classified was the absence of any key term in the text. Records without any categories represented between 6.3 and 15.0%, depending on the species and age class.

**Fig. 3:**
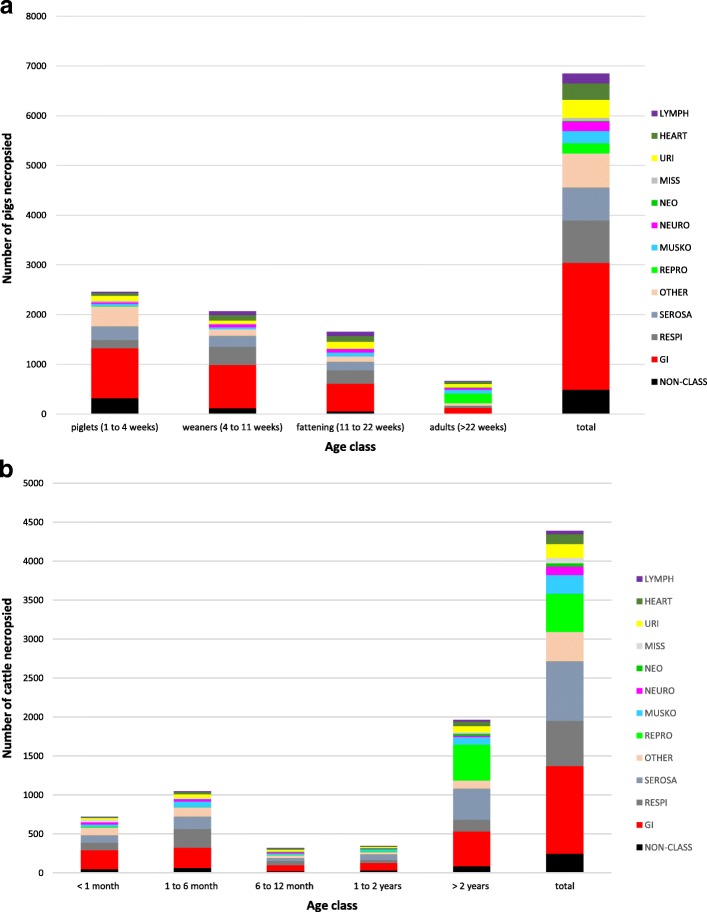
Total number of animals classified into each syndrome by their age classes (the classification numbers are higher than the total number of necropsies because more than one syndrome class per report was possible), based on the automatic categorization of necropsy reports using a text-mining tool with data collected at the Animal Pathology Institute (ITPA) of the University of Bern, Switzerland in 2000–2011. Panel A presents pigs, panel B presents cattle

The proportion of each syndrome varied between species and age categories (Fig. 3). In pigs, respiratory syndromes (69%) were most common in animals greater than 2 years old. Heart (19.8%) and urinary (20.7%) syndromes were especially frequent in pigs between 6 months and 1 year of age. Musculoskeletal syndromes were found in 35.4% of pigs between 1 and 2 years of age, but the number of pigs in this age group was very small.

The most common syndrome in calves less than 1 month of age was GI (54.0%). Gastrointestinal syndromes were important across all age groups of cattle, but to a lesser extent in older age groups. In calves between 1 and 12 months, gastrointestinal (43.0%) and respiratory (34.0%) syndromes were the most common. In cattle between 1 and 2 years of age, the proportion of cattle classified as having a respiratory syndrome decreased to less than 20.0%. Reproductive syndromes were most important in cattle 2 years old or older, where they represented 33.5% of the submissions.

### Time series analysis

There was a decreasing trend in the number of pigs necropsied per year at the ITPA between the years 2000 and 2011. The number of cattle necropsied at the ITPA per year remained stable over the same period (Fig. 1.c1 for pigs and 2.c1 for cattle).

**Fig. 4:**
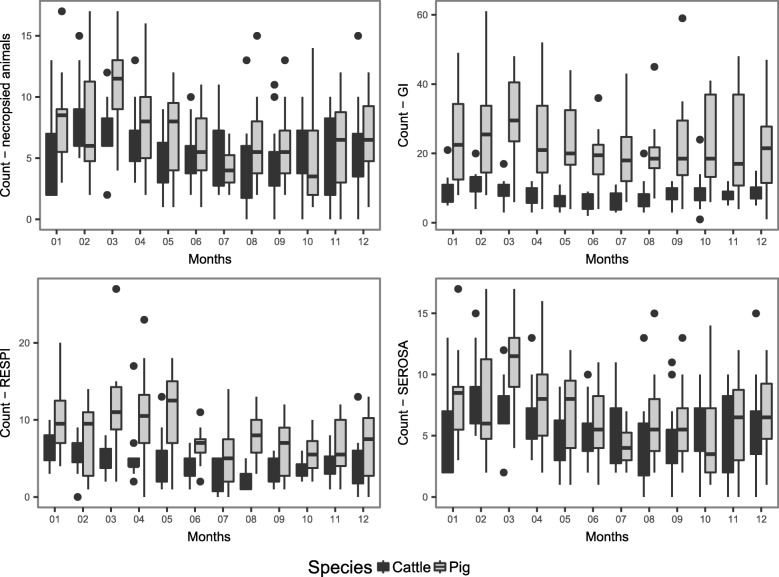
Temporal pattern of the three main syndromes (GI-Gastrointestinal system; RESPI-respiratory system; SEROSA-serous membrane system) of pigs and cattle submissions to the Animal Pathology Institute (ITPA) of the University of Bern, Switzerland in 2000–2012. The count scale is differs among the panels

The number of submissions for both species was lower during summer (June and July) than winter months (January and February; Fig. 4). The number of submissions for both species was also affected by the day of the week. Fewer animals were necropsied during the weekend and more on Mondays (data not shown). For both species, the three main syndromes showed a seasonal trend that mirrored the seasonal trend in the total number of submissions.

## Discussion

In this study, we evaluated the quality of data extracted post-mortem reports from a veterinary diagnostic pathology laboratory and assessed their potential value for animal health surveillance. Extracting data from the text records was relatively easy. Data completeness and validity varied a lot depending on the type of data. Geographical information and personal information such as name and address were present and considered to be valid in most of the reports (i.e., less than 9% of owner addresses and less than 1% of zip codes were unusable), making the data suitable for spatio-temporal analyses and traceability [45]. This information represents an added value that is not often found in other animal health data sources [4]. In contrast, the clinical history was missing in 99% of the necropsy reports. Collecting reliable information on clinical histories could be of great value for animal health surveillance and should be considered for improving the overall value of diagnostic laboratory data. Processes to ensure the quality and reliability of the clinical history reported would also be helpful because clinical histories ranged from owner observations without veterinary consultation to detailed clinical diagnoses made by veterinary specialists (data not shown). Twenty-seven percent of the ages (for pigs and cattle) and 80% of the TVD numbers (for cattle) were also unusable because of missing or invalid values. Poor recording of animal age may limit the use of these data for individual monitoring of different age groups. The TVD number could be used to link necropsy reports to the nationwide animal movement database and track the origin of cases necropsied at ITPA [46]. Improving the recording of these two parameters may thus offer additional opportunities for using the necropsy reports. The most commonly reported problems with the recording of TVD numbers were missing numbers at the time of submission and typing errors when multiple-digit numbers were entered into the computer system. A potential solution to reduce these errors would be to implement an automatic voice recognition system, as already shown to be effective in other fields [47, 48]. In addition, barcode scanners could be used to record the TVD number present on Swiss cattle ear tags and to store it directly in the database [49]. Another potential method to reduce errors would be to implement completeness and correctness checks in the electronic forms that are used for data entry.

The profile of animals necropsied at ITPA was compared to the overall Swiss population to assess the representativeness of these data. In cattle, the balanced sex ratio in young age classes and the preponderance of females in adults was consistent with the Swiss cattle population. Over half (53%) of the cattle necropsied in the study period were less than 6 months of age, which differs from much the smaller proportion of young cattle in the Swiss cattle population [50]. Explanations for this could be a generally higher risk of morbidity and mortality in young animals, or there could be greater difficulty in reaching an on farm clinical diagnosis in young animals. An additional explanation could be that the predominant health problems in adult cattle, such as mastitis, reproductive failure, dystocia and hoof problems typically do not require post-mortem diagnoses. Finally, farmers frequently opt to slaughter an older diseased animal to retain the value of its meat, rather than submitting it for necropsy and further diagnostics. It is not surprising to have differences between the living population and the necropsied population. For a better assessment of the data representativeness, it would be necessary to compare information from necropsy reports to all-cause-mortality data. This data does not exist for pigs in Switzerland, and for cows, the data is only available since 2010. Even though these data have deficiencies, they may still have value for animal health surveillance. There are many factors that might influence the submission (or not) of dead livestock for necropsy. These include factors that affect the behavior of farmers and veterinarians including the economic or sentimental value of the animal, cost of the post mortem examination, postmortem skills of the veterinarian, confidence of the veterinarian in the diagnostic laboratory and others. However, the characteristics of the disease also can influence the submission of dead animal for necropsy. We expect that important diseases that are clinically unusual and the cause significant morbidity and mortality would be submitted to a diagnostic laboratory, and that these rather important diseases should be contained within the data. Monitoring these over time could help to identify new disease emergences.

The seasonality of cattle submitted for necropsy was similar to the seasonality of reported on-farm deaths in Switzerland [17]. We were unable to make the same comparisons for pigs because information to make the comparison was not available.

The ITPA reports did not cover the whole of Switzerland, and the catchment areas for cattle and pigs were clearly centered on the geographic location of the ITPA. This finding is likely due to there being other diagnostic services available to the farmers and the travel distance, as farmers have to transport their animals to the ITPA for necropsy. As expected, as the distance from the farm to Bern increased, the number of necropsied animals decreased. For production animals, there are four additional laboratories, offering necropsy diagnostics in Switzerland. Furthermore, in Switzerland veterinarians commonly perform on farm necropsies especially in the case of small piglets, which can be easily disposed of by the farmer following the necropsy. Additional studies from other laboratories in Switzerland could be performed to see if similar biases are present in other laboratories.

The number of cattle submitted per year remained stable over the 12 years of the study period (200 to 2011), however the number of pigs submitted to the ITPA continuously declined. The reason for the decline in pig submissions could not be determined in this study. Submissions to diagnostic laboratories have been reported to be influenced not only by disease trends, but also by the economic situation in the meat market and other factors [51, 52]. If the reduction in submissions could be determined to be caused by non-disease related factors, it would be a cause of concern if the diagnostic pathology data were used in a national animal health surveillance system. Additional work is needed to determine whether the reduction in swine submissions observed in this study was due to a decline in disease or to other factors. However, these variations could also be of value for animal health surveillance, as investigating them may help improve the understanding of farmer and veterinarian practices, which is essential for building efficient surveillance programs.

The text-mining tool used in this study allowed us to identify pathological patterns in the study population of ITPA necropsy reports. Respiratory and gastrointestinal syndromes were the most common categories identified in both species, which is consistent with previous studies reporting morbidity, mortality and affected organ systems of pigs and cattle in Switzerland, Germany and the USA [53–58]. As expected, reproductive syndromes were most common in animals of reproductive age for both species. Ten percent of the submissions were not classified into a specific syndrome category. There were two types of non-classified animals: animals with an unclear cause of death, and animals that were misclassified (i.e., they should have been classified into at least one syndrome category but the text-mining tool failed to correctly classify them due to misinterpretation). The proportion of misclassified reports (906/8859) was estimated to be low in this dataset, and these cases were mainly caused by undetected negations in the text [36]. However, the number of misclassified reports should be reduced as much as possible to ensure that data is a valid representation of the pathologists’ reports. More standardized procedures for data collection (e.g., avoiding negated diagnoses in the reports) could help to improve the classification performance of the text-mining tool and reduce bias in the data. Monitoring truly unclassified reports might be of value for animal health surveillance, as deaths caused by unknown pathogens might cause an increase in the number of reports in this category [25, 26].

The syndrome categories used in this study were primarily based on the topography of organ systems. The categories chosen are suitable for a wide range of situations and especially for non-specific health monitoring. However, monitoring more specific disease conditions can have advantages. For example, abortions could be identified as a separate syndrome category instead of grouping them into the category reproductive disorders [59], or combining respiratory syndromes and reproductive disorders in pigs might help to monitor Porcine Reproductive & Respiratory Syndrome (PRRS). On the other hand, the more key terms that are grouped into one category, the fewer specific changes that can be detected within that category. For example, the category “other” covers a considerable number of key terms related to a broad range of topographical regions. An increase in a specific pathology, such as conjunctivitis or mastitis, might therefore be masked by the background noise in this category. Thus, further classifications could be necessary, depending on the surveillance objective and its required sensitivity. The text classifying tool used in this study was designed to be highly flexible [36] which means that it is possible to easily modify the syndrome categories in any way that fits with the data and the surveillance objectives. The syndromes that should be used and their characteristics should be determined by the specific goals of the surveillance system. So for example a surveillance system that has the goal of early detection of unusual, previously unseen disease could be based upon a syndrome or set of syndromes that include cases that were classified into the “other” category. These would include cases for which pathologists could not establish a pathological diagnosis [60].

## Conclusion

In this study we have demonstrated that data can be extracted from free text necropsy reports, and that it could potentially be of value for animal health surveillance. The most frequent syndrome categories per species, sex and age, and their respective temporal patterns could serve as a basis for implementing a syndromic surveillance system. This information would not have been obtained without the use of text classification tool. While these methods and necropsy data have potential, the data does have biases and there are improvements that should be made to improve the validity of the data if their value is to be fully realized.

## Additional file

Additional file 1: The table contains the terminology that was used for classification of the reports into syndromic categories. It applies to necropsy reports of cattle and pigs, collected from 2000 to 2011 at the Animal Pathology Laboratory (ITPA), Vetsuisse Faculty, University of Bern. (DOCX 75 kb)

## Abbreviations

AGIS: Agrarpolitical Information System
AI: Artificial insemination
FSVO: Federal Food Safety Authority
ICD: International Classification of Diseases
ITPA: Animal Pathology Institute University of Bern, Switzerland
NLP: Natural language processing
TVD: Swiss animal movement database (Tierverkehrsdatenbank)
