# Supplementary material for: The value of necropsy reports for animal health surveillance
Source: BMC Vet Res. 2018 Jun 18;14:191. doi: 10.1186/s12917-018-1505-1 (PMC6006731; doi:10.1186/s12917-018-1505-1)
Supplement: Supplementary file 1 — The table contains the terminology that was used for classification of the reports into syndromic categories. It applies to necropsy reports of cattle and pigs, collected from 2000 to 2011 at the Animal Pathology Laboratory (ITPA), Vetsuisse Faculty, University of Bern. (DOCX 75 kb) [file 12917_2018_1505_MOESM1_ESM.docx]

| **Syndrome -categorization** | **Word** | **Word variants used** | **Frequency in reports** |
| --- | --- | --- | --- |
| GI | Labmagen-Verlagerung | Labmagen-Verlagerung | 3 |
| GI | Labmagendrehung | Labmagendrehung | 4 |
| GI | Labmagentorsion | Labmagentorsion | 9 |
| GI | Labmagenverlagerung | Labmagenverlagerung | 46 |
| GI | Pansenacidose | Pansenazidose, Pansenacidose | 57 |
| GI | Cheilitis | Cheilitis | 7 |
| GI | Cholangiohepatitis | cholangiohepatitis, Cholangiohepatitis | 18 |
| GI | Cholangiocystitis | Cholangiocystitis | 1 |
| GI | Cholangitis | cholangitis, Cholangitis | 64 |
| GI | Cholecystitis | Cholecystitis, Cholezystitis | 21 |
| GI | Gallengangsfibrose | Gallengangsfibrose | 3 |
| GI | Pericholangitis | pericholangitis, Pericholangitis | 15 |
| GI | Colitis | kolitis, colitis, Colitis, Kolitis | 420 |
| GI | Blinddarmdilatation | Blinddarmdilatation | 1 |
| GI | Labmagen-Dilatation | Labmagen-Dilatation | 2 |
| GI | Labmagendilatation | Labmagendilatation | 14 |
| GI | Magendilatation | Magendilatation | 4 |
| GI | Magenüberladung | Magenüberladung | 7 |
| GI | Pansen-Tympanie | Pansen-Tympanie, Pansen-Tympanien | 10 |
| GI | Pansendilatation | Pansendilatation | 8 |
| GI | Pansentympanie | Pansentympanie | 10 |
| GI | Tympanie | tympani, Tympanie, Tympanien | 138 |
| GI | tympany | tympany | 3 |
| GI | Duodenitis | duodenitis, Duodenitis | 15 |
| GI | -Diarrhoe | -Diarrhoe | 1 |
| GI | Darmkokzidiose | Darmkokzidiose | 6 |
| GI | Diarrhea | Diarrhea, diarrhea | 6 |
| GI | Diarrhoe | Diarrhoe, diarrhoe | 232 |
| GI | Diarrhöe | Diarrhöe | 40 |
| GI | Diarrhoë | Diarrhoë | 2 |
| GI | Druchfalls | Druchfalls | 1 |
| GI | Durchall | Durchall | 1 |
| GI | Durchfall | Durchfall, Durchfalls, Durchfalles | 287 |
| GI | Durchfall-Erkrankungen | Durchfall-Erkrankungen | 1 |
| GI | Durchfall-Ursache | Durchfall-Ursache | 3 |
| GI | Durchfallerkrankung | Durchfallerkrankung | 5 |
| GI | Durchfallserkrankung | Durchfallserkrankung | 1 |
| GI | Durchfallsursache | Durchfallsursache | 3 |
| GI | Durchfallursache | Durchfallursachen, Durchfallursache | 43 |
| GI | Durfallursache | Durfallursache | 1 |
| GI | Endoparasitose | Endoparasitose | 25 |
| GI | Enterolith | Enterolith, Enterolithen | 2 |
| GI | Enteropathie | Enteropathie, enteropathie | 78 |
| GI | enteropathy | enteropathy | 4 |
| GI | Enteropatie | Enteropatie | 1 |
| GI | Enterophathie | Enterophathie | 1 |
| GI | Gastroenteritis | Gastroenteritis | 10 |
| GI | Gastroenterokolitis | Gastroenterokolitis, gastroenterocolitis | 4 |
| GI | C-Enteritis | C-Enteritis | 48 |
| GI | Kälberdurchfall | Kälberdurchfall | 3 |
| GI | Coil-Diarrhoe | Coil-Diarrhoe | 1 |
| GI | Kokkzidiose | Kokkzidiose | 4 |
| GI | Kokzidiode | Kokzidiode | 1 |
| GI | Coccidiose | Kokzidiose, Coccidiose | 183 |
| GI | Kokzidose | Kokzidose | 2 |
| GI | Coli-Diarrhoe | coli-Diarrhoe, Coli-Diarrhoe, Coli-diarrhoe | 784 |
| GI | Coli-Diarrhöe | Coli-Diarrhöe | 32 |
| GI | Coli-Diarrhoë | Coli-Diarrhoë | 1 |
| GI | Coli-Diarrrhoe | Coli-Diarrrhoe | 1 |
| GI | Coli-Durchfall | Coli-Durchfalles, coli-Durchfall, Coli-Durchfall | 21 |
| GI | Colidarrhoe | Colidarrhoe | 2 |
| GI | Colidiarrhoe | Colidiarrhoe | 74 |
| GI | Colidiarrhöe | Colidiarrhöe | 9 |
| GI | Colidurchfall | Colidurchfall | 2 |
| GI | Colienteritis | Colienteritis | 1 |
| GI | Corona-Virus-Diarrhoe | Corona-Virus-Diarrhoe | 1 |
| GI | Coronavirus-Diarrhoe | Coronavirus-Diarrhoe | 1 |
| GI | Cryptosporidien-Diarrhoe | Cryptosporidien-Diarrhoe | 1 |
| GI | Cryptosporidiendiarrhoe | Cryptosporidiendiarrhoe | 1 |
| GI | Cryptosporidiose | Kryptosporidiose, Cryptosporidiose | 39 |
| GI | Kryptosporridiose | Kryptosporridiose | 1 |
| GI | Rota-Coronavirus-Diarrhoe | Rota-Coronavirus-Diarrhoe | 1 |
| GI | Rota-Coronavirus-Durchfall | Rota-Coronavirus-Durchfall | 1 |
| GI | Rota-Virus-Diarrhoe | Rota-Virus-Diarrhoe | 1 |
| GI | Rota-Virus-Infektion | Rota-Virus-Infektion | 1 |
| GI | Rota-Virusinfektion | Rota-Virusinfektion | 1 |
| GI | Rotavireninfektion | Rotavireninfektion | 1 |
| GI | Rotavirose | Rotavirose | 5 |
| GI | Rotavirus-Diarrhoe | Rotavirus-Diarrhoe | 6 |
| GI | Rotavirus-Durchfall | Rotavirus-Durchfall | 2 |
| GI | Rotavirus-Enteritis | Rotavirus-Enteritis | 12 |
| GI | Rotavirus-Infektion | Rotavirus-Infektion | 7 |
| GI | Rotavirusdiarrhoe | Rotavirusdiarrhoe | 2 |
| GI | Rotavirusinfektion | Rotavirusinfektion | 14 |
| GI | Salmonella-Infektion | Salmonella-Infektion | 1 |
| GI | Salmonelleninfektion | Salmonelleninfektion | 5 |
| GI | Salmonellose | Salmonellose | 10 |
| GI | Typ-C-Enteritis | Typ-C-Enteritis | 1 |
| GI | Virus-Diarrhoe | Virus-Diarrhoe | 5 |
| GI | Virusdiarrhoe | Virusdiarrhoe | 3 |
| GI | Enterotoxämie | Enterotoxaemie, Enterotoxämie | 17 |
| GI | Clostridien-Enterotoxämie | Clostridien-Enterotoxämie, Clostridien-Enterotoxaemie | 5 |
| GI | Clostridienenterotoxämie | Clostridienenterotoxämie | 1 |
| GI | Coli-Enterotoxämie | Coli-Enterotoxämie, Coli-Enterotoxaemie | 277 |
| GI | Coli-Enterotoxamie | Coli-Enterotoxamie | 1 |
| GI | Coli-Enterotoxonie | Coli-Enterotoxonie | 1 |
| GI | Coli-Entertoxaemie | Coli-Entertoxaemie | 1 |
| GI | Colienterotöxämie | Colienterotöxämie | 1 |
| GI | Colienterotoxämie | Colienterotoxämie, Colienterotoxaemie | 5 |
| GI | Gastritis | Gastritis | 153 |
| GI | Kardia-Stenose | Kardia-Stenose | 1 |
| GI | Cardiastenose | Cardiastenose, Kardiastenose | 20 |
| GI | Labmagen Ulzera | Labmagen Ulzera | 8 |
| GI | Magengeschwür | Magengeschwüre, Magengeschwür | 55 |
| GI | Magenulcus | Magenulkus, Magenulcus | 134 |
| GI | Magenulcera | Magenulzera, Magenulcera | 39 |
| GI | Magenulzeration | Magenulzerationen, Magenulzeration | 9 |
| GI | perforierte Ulcus | perforierter Ulkus, perforierte Ulcus, perforierter Ulcus, perforiertem Ulcus, perforierten Ulkus, perforiertes Ulcus, Perforiertes Ulkus, perforiertem Ulkus, perforierten Ulcus, perforiertes Ulkus | 36 |
| GI | -hepatitis | -hepatitis | 1 |
| GI | Hepatitis | Hepatitis, hepatitis | 203 |
| GI | Hepatose | Hepatose | 1 |
| GI | Hepatosis | Hepatosis | 3 |
| GI | Perihepatits | Perihepatits | 1 |
| GI | Perihepatitis | Perihepatitis | 17 |
| GI | Abdominalhernie | Abdominalhernie | 2 |
| GI | Hiatushernie | Hiatushernie | 4 |
| GI | Inguinalhernie | Inguinalhernie | 18 |
| GI | Zwerchfellhernie | Zwerchfellhernie | 5 |
| GI | Zwerchfellshernie | Zwerchfellshernie | 3 |
| GI | Hämorrhagisches Intestinal Syndrom | Hämorrhagisches intestinales Syndrom, Hämorrhagische Intestinale Syndrom, Hämorrhagischen Intestinal Syndrom, Hämorrhagisches Intestinales Syndrom, Hämorrhagisches Intestinal Syndrom, Hämorrhagischen Intestinalen Syndrom | 12 |
| GI | HIS | HIS, His | 83 |
| GI | Intestinal-Syndrom | Intestinal-Syndrom | 1 |
| GI | Intestinalesyndrom | Intestinalesyndrom | 2 |
| GI | Intestinalsyndrom | Intestinalsyndrom, intestinalsyndrom | 350 |
| GI | Intestinalsynsdrom | Intestinalsynsdrom | 1 |
| GI | Hoflund | Hoflund | 5 |
| GI | Hoflund-Syndrom | Hoflund-Syndrom | 4 |
| GI | Hoflundsyndrom | Hoflundsyndrom | 2 |
| GI | Ikterus | Ikterus | 64 |
| GI | Ileitis | Ileitis | 67 |
| GI | Ileo-Thyphlo-Colitis | Ileo-Thyphlo-Colitis | 1 |
| GI | Ileothyphlocolitis | Ileothyphlocolitis | 1 |
| GI | Illeitis | Illeitis | 1 |
| GI | Darminvagination | Darminvagination | 1 |
| GI | Invaginatio | Invaginatio | 7 |
| GI | Invagination | Invaginationen, Invagination | 65 |
| GI | Invaginationsstelle | Invaginationsstelle | 2 |
| GI | Coloninvagination | Coloninvagination | 1 |
| GI | Jejunitis | Jejunitis | 33 |
| GI | Labmagen-Erosionen | Labmagen-Erosionen | 1 |
| GI | Labmagen-Ulzera | Labmagen-Ulzera | 1 |
| GI | Labmagen-Ulzerationen | Labmagen-Ulzerationen | 1 |
| GI | Labmagenerosion | Labmagenerosion, Labmagenerosionen | 3 |
| GI | Labmagengeschwür | Labmagengeschwüre, Labmagengeschwür | 4 |
| GI | Labmagenulcus | Labmagenulkus, Labmagenulcus | 69 |
| GI | Labmagenulcera | Labmagenulzera, Labmagenulcera | 86 |
| GI | Labmagenulzeration | Labmagenulzeration, Labmagenulzerationen | 46 |
| GI | Fettleber | Fettleber | 16 |
| GI | Leberlipidose | Leberlipidose | 3 |
| GI | Leberverfettung | Leberverfettung | 22 |
| GI | Lipidose | Lipidose, Lipidosen | 43 |
| GI | Lipidoseherde | Lipidoseherde | 1 |
| GI | Lipidosis | Lipidosis, lipidosis | 14 |
| GI | Steatose | Steatose | 18 |
| GI | Steatosis | Steatosis | 2 |
| GI | Koprostase | Koprostase | 7 |
| GI | Obstipation | Obstipation | 7 |
| GI | Obstipationsstelle | Obstipationsstelle | 1 |
| GI | Periesophagitis | Periesophagitis | 1 |
| GI | Periösophagitis | Periösophagitis, Perioesophagitis | 6 |
| GI | Pankreatitis | Pankreatitis | 3 |
| GI | Makrogamonten | Makrogamonten | 10 |
| GI | Monezia |  |  |
| GI | Zestoden | Zestoden, Cestoden | 5 |
| GI | Darmperforation | Darmperforation | 7 |
| GI | Darmwandperforationen | Darmwandperforationen | 2 |
| GI | Dünndarmperforation | Dünndarmperforation | 1 |
| GI | Duodenum-Perforation | Duodenum-Perforation | 2 |
| GI | Jejunumperforation | Jejunumperforation | 1 |
| GI | Colonperforation | Colonperforation, Kolonperforation | 5 |
| GI | Labmagenperforation | Labmagenperforation | 8 |
| GI | Mesenterialperforation | Mesenterialperforation | 1 |
| GI | Ösophagusperforation | Ösophagusperforation | 3 |
| GI | Pansenperforation | Pansenperforation | 3 |
| GI | Rektumperforation | Rektumperforation | 1 |
| GI | Rektumsperforation | Rektumsperforation | 1 |
| GI | Schleimhautperforation | Schleimhautperforation | 2 |
| GI | Caecumperforation | Caecumperforation, Caecumperforationen | 2 |
| GI | Zäkumsperforation | Zäkumsperforation | 1 |
| GI | Pharyngitis | Pharyngitis | 10 |
| GI | Proctitis | Proktitis, Proctitis, proctitis | 17 |
| GI | Reticulitis | Retikulitis, reticulitis, Reticulitis | 29 |
| GI | Retikulo-Perikarditis | Retikulo-Perikarditis | 1 |
| GI | Reticulo-Peritonitis | reticulo-peritonitis, Reticulo-Peritonitis, Retikulo-Peritonitis | 12 |
| GI | Reticulopericarditis | reticulopericarditis, Reticulopericarditis | 3 |
| GI | Reticuloperitonitis | reticuloperitonitis, Retikuloperitonitis, Reticuloperitonitis | 71 |
| GI | Retiukuloperitonitis | Retiukuloperitonitis | 1 |
| GI | Sialoadenitis | Sialoadenitis | 2 |
| GI | Darmstenose | Darmstenose | 2 |
| GI | Dünndarmstenose | Dünndarmstenose | 5 |
| GI | Rectumstenose | Rectumstenose, Rektumstenose | 4 |
| GI | Staetorrhoe | Staetorrhoe | 1 |
| GI | Steatorhoe | Steatorhoe | 1 |
| GI | Steatorrhea | Steatorrhea | 1 |
| GI | Steatorrhoe | Steatorrhoe | 106 |
| GI | Steatorrhöe | Steatorrhöe | 3 |
| GI | Darmperforation | Darmperforation | 7 |
| GI | Darmwandperforationen | Darmwandperforationen | 2 |
| GI | Dünndarmperforation | Dünndarmperforation | 1 |
| GI | Ileumruptur | Ileumruptur | 1 |
| GI | Labmagenruptur | Labmagenruptur | 7 |
| GI | Magenruptur | Magenruptur | 7 |
| GI | Magenwandruptur | Magenwandruptur | 1 |
| GI | Pansenverletzung | Pansenverletzung | 1 |
| GI | Retikulo-Peritonitis-traumatica | Retikulo-Peritonitis-traumatica | 1 |
| GI | Thyphlitis | Thyphlitis | 7 |
| GI | Tiphlitis | Tiphlitis | 1 |
| GI | Typhlitis | Typhlitis, typhlitis | 34 |
| GI | Darmtorsion | Darmtorsion | 21 |
| GI | Darmtrakt-Volvulus | Darmtrakt-Volvulus | 1 |
| GI | Darmvolvulus | Darmvolvulus | 2 |
| GI | Dickdarmtorsion | Dickdarmtorsion | 1 |
| GI | Dünndarmdrehungen | Dünndarmdrehungen | 1 |
| GI | Dünndarmtorsion | Dünndarmtorsion | 10 |
| GI | Dünndarmvolvulus | Dünndarmvolvulus | 5 |
| GI | Jejunuminkarzeration | Jejunuminkarzeration | 1 |
| GI | Colonscheibentorsion | Colonscheibentorsion | 4 |
| GI | Colontorsion | Kolontorsion, Colontorsion | 10 |
| GI | Labmagenvolvulus | Labmagenvolvulus | 1 |
| GI | Magentorsion | Magentorsion | 1 |
| GI | Torsio mesenteralis | Torsio mesenteralis | 1 |
| GI | Torsio mesenterialis | Torsio mesenterialis | 12 |
| GI | Torsion der Gekrösewurzel | Torsion der Gekrösewurzel | 32 |
| GI | Torsion der Zäkumspitze | Torsion der Zäkumspitze | 1 |
| GI | Torsion des Dünndarmkonvoluts | Torsion des Dünndarmkonvolutes | 2 |
| GI | Torsion des Mesenteriums | Torsion des Mesenteriums | 4 |
| GI | Voluvlus | Voluvlus | 1 |
| GI | Volvolus | Volvolus | 19 |
| GI | Volvulus | Volvulus, volvulus | 484 |
| GI | Vovulus | Vovulus | 1 |
| GI | Caecum-Torsion | Caecum-Torsion | 3 |
| GI | Zäkumstorsion | Zäkumstorsion | 1 |
| GI | Zäkumtorsion | Zäkumtorsion, Caecumtorsion | 7 |
| GI | Abomasitis | abomasitis, Abomasitis | 160 |
| GI | Anomasitis | Anomasitis | 1 |
| GI | Glossitis | Glossitis, glossitis | 19 |
| GI | Esophagitis | Esophagitis | 2 |
| GI | Oesophagiits | Oesophagiits | 1 |
| GI | Ösophagitis | Oesophagitis, Ösophagitis | 47 |
| GI | Omasitis | Omasitis, omasitis | 24 |
| GI | Pansenulkus | Pansenulkus | 1 |
| GI | Pansenulzera | Pansenulzera | 1 |
| GI | Pansenulzeration | Pansenulzeration, Pansenulzerationen | 3 |
| GI | Rumenitis | rumenitis, Rumenitis | 93 |
| GI | Rumenitits | Rumenitits | 1 |
| GI | Rumentitis | Rumentitis | 1 |
| GI | Ruminitis | Ruminitis, ruminitis | 24 |
| GI | Rumitis | Rumitis | 1 |
| GI | Gingivitis | Gingivitis, gingivitis | 9 |
| GI | Perigingivitis | Perigingivitis | 1 |
| GI | Stomatiden | Stomatiden | 1 |
| GI | Stomatits | Stomatits | 1 |
| GI | Stomatitiden | Stomatitiden | 1 |
| GI | Stomatitis | stomatitis, Stomatitis | 60 |
| GI | Thyphlocolitis | Thyphlokolitis, Thyphlocolitis | 6 |
| GI | Typhlocolitis | Typhlocolitis, Typhlokolitis | 96 |
| GI | Typhocolitis | Typhocolitis | 1 |
| GI | Enterocolitis | Enterokolitis, Enterocolitis, enterocolitis | 206 |
| GI | Thyphlocolitis | Thyphlokolitis, Thyphlocolitis | 6 |
| GI | Enetritis | Enetritis | 1 |
| GI | Enteritiden | Enteritiden | 1 |
| GI | Enteritidis | Enteritidis | 1 |
| GI | Enteritis | enteritis, Enteritis | 1093 |
| GI | Enteritis-Kolitis | Enteritis-Kolitis | 1 |
| GI | Enteritits | Enteritits | 1 |
| GI | Entero-Colitis | Entero-Colitis | 1 |
| GI | Entero-Typhlokolitis | Entero-Typhlokolitis | 3 |
| GI | Enterotyphlitis | Enterotyphlitis | 1 |
| GI | Enterotyphlocolitis | Enterotyphlocolitis, Enterotyphlokolitis | 24 |
| GI | Entertitis | Entertitis | 1 |
| GI | Entreritis | Entreritis | 1 |
| HEART | Maulbeer-Herzkrankheit | Maulbeer-Herzkrankheit | 1 |
| HEART | Maulbeerherz | Maulbeerherz | 1 |
| HEART | Maulbeerherz-Krankheit | Maulbeerherz-Krankheit | 1 |
| HEART | Maulbeerherzkrankheit | Maulbeerherzkrankheit | 13 |
| HEART | Mikroangiopathie | Mikroangiopathie | 11 |
| HEART | Microangiopathia | Microangiopathia, Mikroangiopathia | 50 |
| HEART | Aortenklappen-Endokarditis | Aortenklappen-Endokarditis | 1 |
| HEART | Endokarditiden | Endokarditiden | 1 |
| HEART | Endocarditis | Endokarditis, endocarditis, Endocarditis | 257 |
| HEART | Endokartitis | Endokartitis | 1 |
| HEART | Endokraditis | Endokraditis | 1 |
| HEART | Mitralklappenendokarditis | Mitralklappenendokarditis | 1 |
| HEART | Streptokokken-Endokarditis | Streptokokken-Endokarditis | 1 |
| HEART | Trikuspidalendokarditis | Trikuspidalendokarditis | 1 |
| HEART | Endokardiose | Endokardiose | 12 |
| HEART | Endocardiosis | Endocardiosis, endocardiosis | 2 |
| HEART | Herzinfarkt | Herzinfarkt | 3 |
| HEART | Myokard-Degeneration | Myokard-Degeneration | 5 |
| HEART | Myokarddegeneration | Myokarddegeneration | 16 |
| HEART | Myokardfaserdegeneration | Myokardfaserdegeneration | 4 |
| HEART | Myokardfaserndegeneration | Myokardfaserndegeneration | 1 |
| HEART | Myokardialnekrosen | Myokardialnekrosen | 1 |
| HEART | Myokardnekrose | Myokardnekrose, Myokardnekrosen | 18 |
| HEART | Myokardzellnekrose | Myokardzellnekrose | 1 |
| HEART | hidropericardium | hidropericardium | 1 |
| HEART | Hydroperikadium | Hydroperikadium | 1 |
| HEART | Hydropericard | Hydropericard, Hydroperikard | 318 |
| HEART | Hydroperikardium | Hydroperikardium | 8 |
| HEART | Hypdroperikard | Hypdroperikard | 1 |
| HEART | Herz-Kreislaufinsuffizienz | Herz-Kreislaufinsuffizienz | 2 |
| HEART | Herzhypertrophie | Herzhypertrophie | 2 |
| HEART | Herzinsuffisienz | Herzinsuffisienz | 1 |
| HEART | Herzinsuffizienz | Herzinsuffizienz | 20 |
| HEART | Herzkreislaufinsuffizienz | Herzkreislaufinsuffizienz | 1 |
| HEART | Cardiomyopathie | Kardiomyopathie, Cardiomyopathie, Kardiomyopathien | 25 |
| HEART | Kardiomyophathie | Kardiomyophathien, Kardiomyophathie | 2 |
| HEART | Kardiomypathie | Kardiomypathie | 1 |
| HEART | Linksherzinsuffizienz | Linksherzinsuffizienz | 4 |
| HEART | Myokardhypertrophie | Myokardhypertrophie | 1 |
| HEART | Myokardiopathie | Myokardiopathie | 1 |
| HEART | Rechtsherzhypertrophie | Rechtsherzhypertrophie | 2 |
| HEART | Rechtsherzinsuffisuzienz | Rechtsherzinsuffisuzienz | 1 |
| HEART | Rechtsherzinsuffizienz | Rechtsherzinsuffizienz | 4 |
| HEART | Myokardits | Myokardits | 1 |
| HEART | Myocarditis | Myokarditis, myocarditis, Myocarditis | 93 |
| HEART | Myokarditits | Myokarditits | 1 |
| LYMPH | Lymphknotenveränderungen | Lymphknotenveränderungen | 2 |
| LYMPH | Lymphomegalie | Lymphomegalie | 1 |
| LYMPH | Milztorison | Milztorison | 1 |
| LYMPH | Milztorsion | Milztorsion | 25 |
| LYMPH | Perisplenitis | Perisplenitis | 7 |
| LYMPH | Splenitis | Splenitis | 75 |
| LYMPH | Lymphadenis | Lymphadenis | 1 |
| LYMPH | Lymphadenits | Lymphadenits | 1 |
| LYMPH | Lymphadeniti | Lymphadeniti | 2 |
| LYMPH | Lymphadenitis | Lymphadenitis, lymphadenitis | 257 |
| LYMPH | Lymphadenitits | Lymphadenitits | 1 |
| LYMPH | Lymphadenomegalie | Lymphadenomegalie | 20 |
| LYMPH | lymphadentits | lymphadentits | 1 |
| LYMPH | lymphandenitis | lymphandenitis | 1 |
| LYMPH | Tonsilitis | Tonsilitis | 4 |
| LYMPH | Tonsillitis | Tonsillitis | 93 |
| MISS | Schistosoma | Schistosoma | 2 |
| MISS | Atresie | Atresie | 8 |
| MISS | Atresia | Atresia | 26 |
| MISS | Darmektopie | Darmektopie | 1 |
| MISS | Hemiatresie | Hemiatresie | 1 |
| MISS | Aortenklappendysplasie | Aortenklappendysplasie | 1 |
| MISS | Atrialseptumdefekt | Atrialseptumdefekt | 1 |
| MISS | Atriumseptumdefekt | Atriumseptumdefekt | 2 |
| MISS | Botalli | botalli, Botalli | 33 |
| MISS | foramen ovale persistens | Foramen ovale persistens | 8 |
| MISS | Herzmissbildung | Herzmissbildung, Herzmissbildungen | 27 |
| MISS | Hoch-Ventrikelseptumdefekt | Hoch-Ventrikelseptumdefekt | 3 |
| MISS | Persistierende Duktus arteriosus | persistierender Ductus arteriosus, Persistierende Duktus arteriosus, persistierenden Ductus arteriosus | 3 |
| MISS | persistierendes foramen ovale | Persistierendes Foramen Ovale, Persistierende Foramen ovale | 2 |
| MISS | Septen-Defekt | Septen-Defekt | 1 |
| MISS | Septumdefekt | Septumdefekte, Septumdefekts, Septumdefekt | 39 |
| MISS | Septumsdefekt | Septumsdefekt | 4 |
| MISS | Venrikel-Septum-Defekt | Venrikel-Septum-Defekt | 1 |
| MISS | Ventrikel-Septum-Defekt | Ventrikel-Septum-Defekt | 7 |
| MISS | Ventrikel-Septumdefekt | Ventrikel-Septumdefekt | 2 |
| MISS | Ventrikelseptumdefekt | Ventrikelseptumdefekt, Ventrikelseptumdefekts, Ventrikelseptumdefektes | 60 |
| MISS | Ventrikelseptumsdefekt | Ventrikelseptumsdefekt | 3 |
| MISS | ventricular septal defect | ventricular septal defect, Ventricular septal defect | 3 |
| MISS | Vorhofseptum defekt | Vorhofseptum defekt | 1 |
| MISS | Vorhofseptumdefekt | Vorhofseptumdefekt | 7 |
| MISS | Arthrogrypose | Arthrogrypose | 10 |
| MISS | Arthrogryposis | arthrogryposis, Arthrogryposis | 11 |
| MISS | Athrogryposis | Athrogryposis | 1 |
| MISS | bifida | bifida | 3 |
| MISS | Brachygnathia | Brachygnathia | 5 |
| MISS | Brachygnatie | Brachygnatie | 2 |
| MISS | Brachygnatia | Brachygnatia | 1 |
| MISS | Brachygnatiea | Brachygnatiea | 1 |
| MISS | Brachygnatismus | Brachygnatismus | 2 |
| MISS | Diplomyelie | Diplomyelie | 2 |
| MISS | Lippen-Gaumenspalte | Lippen-Gaumenspalte | 2 |
| MISS | Malformation | malformations, malformation, Malformation, Malformationen | 11 |
| MISS | Palathoschisis | Palathoschisis | 1 |
| MISS | Palatoschisis | palatoschisis, Palatoschisis | 4 |
| MISS | Prognathie | Prognathie | 2 |
| MISS | Sakrokokzygealgelenksmissbildung | Sakrokokzygealgelenksmissbildung | 1 |
| MISS | Hypomyelinogenese | Hypomyelinogenese | 3 |
| MISS | Kleinhirnhypoplasie | Kleinhirnhypoplasie | 6 |
| MISS | Freemartin | Freemartin | 2 |
| MISS | Freemartinism | Freemartinism | 1 |
| MISS | Freemartinismus | Freemartinismus | 2 |
| MISS | Hodenaplasie | Hodenaplasie | 1 |
| MISS | Pseudohermaphrodismus | Pseudohermaphrodismus | 1 |
| MISS | Pseudohermaphroditismus | Pseudohermaphroditismus | 1 |
| MUSKO | -Arthritis | -Arthritis, -arthritis | 8 |
| MUSKO | Arthitis | Arthitis | 1 |
| MUSKO | Arthristis | Arthristis | 1 |
| MUSKO | Arthritis | arthritis, Arthritis | 232 |
| MUSKO | artritis | artritis | 1 |
| MUSKO | Gonarthritis | Gonarthritis | 7 |
| MUSKO | Gonitis | Gonitis | 18 |
| MUSKO | Hämarthros | Hämarthros, Hämarthrose | 4 |
| MUSKO | hemarthrosis | hemarthrosis | 2 |
| MUSKO | hemearthrosis | hemearthrosis | 1 |
| MUSKO | Coxarthritis | Coxarthritis | 10 |
| MUSKO | Monarthritis | Monarthritis | 4 |
| MUSKO | Monoarthritis | Monoarthritis | 3 |
| MUSKO | MT3-P1arthritis | MT3-P1arthritis | 1 |
| MUSKO | Osteoarthritis | Osteoarthritis | 3 |
| MUSKO | Periarthritis | Periarthritis | 24 |
| MUSKO | Spondylarthritis | Spondylarthritis | 2 |
| MUSKO | Arthrosynovitis | arthrosynovitis, Arthrosynovitis | 4 |
| MUSKO | Arthrose | Arthrose | 16 |
| MUSKO | Arthrosis | Arthrosis | 1 |
| MUSKO | Gonarthrose | Gonarthrose | 1 |
| MUSKO | Hämarthros | Hämarthros, Hämarthrose | 4 |
| MUSKO | hemarthrosis | hemarthrosis | 2 |
| MUSKO | Knorpelerosion | Knorpelerosionen, Knorpelerosion | 14 |
| MUSKO | Knorpelusuren | Knorpelusuren | 4 |
| MUSKO | Coxarthrose | Coxarthrose | 1 |
| MUSKO | Polyarthrosen | Polyarthrosen | 1 |
| MUSKO | Pseudoarthrose | Pseudoarthrose | 1 |
| MUSKO | Usuren | Usuren | 2 |
| MUSKO | Callus | Callus, Kallus | 7 |
| MUSKO | Kallus-Bildung | Kallus-Bildung | 1 |
| MUSKO | Callusbildung | Callusbildung, Kallusbildung | 8 |
| MUSKO | Kallussen | Kallussen | 1 |
| MUSKO | Knochenersatz | Knochenersatz | 1 |
| MUSKO | Knochenkallus | Knochenkallus | 1 |
| MUSKO | Beckenfraktur | Beckenfrakturen, Beckenfraktur | 5 |
| MUSKO | Femurfraktur | Femurfraktur, Femurfrakturen | 2 |
| MUSKO | Femurkopf-Fraktur | Femurkopf-Fraktur | 1 |
| MUSKO | Femurkopffraktur | Femurkopffraktur | 1 |
| MUSKO | Fraktur | Fracture, Frakturen, fractures, Fraktur, fracture | 140 |
| MUSKO | fractured | fractured | 4 |
| MUSKO | frakturiert | frakturierten, frakturiertes, frakturiert, frakturierter | 14 |
| MUSKO | Frakturkallus | Frakturkallus | 1 |
| MUSKO | Frakturlinie | Frakturlinie | 3 |
| MUSKO | Frakturspalt | Frakturspalt | 2 |
| MUSKO | Frakturstelle | Frakturstellen, Frakturstelle | 3 |
| MUSKO | Frakur | Frakur | 1 |
| MUSKO | Infraktion | Infraktion | 2 |
| MUSKO | Querfraktur | Querfraktur | 1 |
| MUSKO | Rippenfraktur | Rippenfrakturen, Rippenfraktur | 15 |
| MUSKO | Schrägfraktur | Schrägfraktur | 7 |
| MUSKO | Splitterfraktur | Splitterfraktur, Splitterfrakturen | 3 |
| MUSKO | Tibiafraktur | Tibiafraktur | 2 |
| MUSKO | Wirbelfraktur | Wirbelfraktur | 3 |
| MUSKO | Hüftgelenksluxation | Hüftgelenksluxation | 3 |
| MUSKO | Luxation | Luxation | 13 |
| MUSKO | Wirbelkörperluxation | Wirbelkörperluxation | 1 |
| MUSKO | Belastungsmyopathie | Belastungsmyopathie | 1 |
| MUSKO | Muskeldegeneration | Muskeldegeneration | 10 |
| MUSKO | Muskelfaserdegeneration | Muskelfaserdegeneration | 3 |
| MUSKO | Muskelfaserndegeneration | Muskelfaserndegeneration | 1 |
| MUSKO | Muskelfasernekrose | Muskelfasernekrose | 1 |
| MUSKO | Muskelläsionen | Muskelläsionen | 2 |
| MUSKO | Muskelnekrose | Muskelnekrose, Muskelnekrosen | 23 |
| MUSKO | Muskelzelldegeneration | Muskelzelldegeneration | 1 |
| MUSKO | Myodegeneration | Myodegeneration, myodegeneration | 7 |
| MUSKO | Myofaserdegeneration | Myofaserdegeneration | 1 |
| MUSKO | Myonekrose | Myonekrose | 1 |
| MUSKO | myonecrosis | myonecrosis | 1 |
| MUSKO | Rhabdomyolyse | Rhabdomyolyse | 1 |
| MUSKO | Weiss-Muskel-Krankheit | Weiss-Muskel-Krankheit | 1 |
| MUSKO | Weiss-Muskelkrankheit | Weiss-Muskelkrankheit | 2 |
| MUSKO | Weissmuskelerkrankung | Weissmuskelerkrankung | 4 |
| MUSKO | Weissmuskelkrankeit | Weissmuskelkrankeit | 2 |
| MUSKO | Weissmuskelkrankheit | Weissmuskelkrankheit | 40 |
| MUSKO | -myositis | -myositis | 1 |
| MUSKO | Myopathie | Myopathie, Myopathien | 5 |
| MUSKO | Myositis | myositis, Myositis | 60 |
| MUSKO | Osteochondritis | Osteochondritis | 2 |
| MUSKO | Osteochondrose | Osteochondrose | 24 |
| MUSKO | Osteochondrosis | Osteochondrosis | 9 |
| MUSKO | osteochondrotische | osteochondrotische | 3 |
| MUSKO | Apophysiolyse | Apophysiolyse | 3 |
| MUSKO | Apophysiolysis | Apophysiolysis | 4 |
| MUSKO | Epiphysiolyse | Epiphysiolyse | 3 |
| MUSKO | Epiphysiolysis | Epiphysiolysis | 8 |
| MUSKO | Osteopenie | Osteopenie | 4 |
| MUSKO | Carpitis | carpitis, Karpitis, Carpitis | 13 |
| MUSKO | Oesteomyelitis | Oesteomyelitis | 2 |
| MUSKO | Oestomyelitis | Oestomyelitis | 1 |
| MUSKO | Ostemyelitis | Ostemyelitis | 1 |
| MUSKO | ostemylitis | ostemylitis | 1 |
| MUSKO | osteomyelitische | osteomyelitische, osteomyelitischer, osteomyelitischen | 4 |
| MUSKO | Osteomyelitis | osteomyelitis, Osteomyelitis | 166 |
| MUSKO | Osteomyelitis-Herd | Osteomyelitis-Herde, Osteomyelitis-Herd | 2 |
| MUSKO | Osteomyelitisherd | Osteomyelitisherd | 2 |
| MUSKO | Osteomylitis | Osteomylitis | 2 |
| MUSKO | Peritarsitis | Peritarsitis | 4 |
| MUSKO | Tarsitis | tarsitis, Tarsitis | 17 |
| MUSKO | Wirbelkörper-Osteomyelitis | Wirbelkörper-Osteomyelitis | 1 |
| MUSKO | Ruptur von Adduktorenmuskeln | Ruptur von Adduktorenmuskeln | 1 |
| MUSKO | Synovitis | synovitis, Synovitis | 24 |
| MUSKO | Tendosynovitis | Tendosynovitis | 12 |
| MUSKO | tenosinovitis | tenosinovitis | 1 |
| MUSKO | Tenosynovitis | Tenosynovitis, tenosynovitis | 15 |
| MUSKO | Tendinitis | Tendinitis | 7 |
| MUSKO | Tendovaginitis | tendovaginitis, Tendovaginitis | 22 |
| MUSKO | Pododermatitis | pododermatitis, Pododermatitis | 23 |
| MUSKO | Sohlengeschwür | Sohlengeschwür | 10 |
| NEO | Adenokarzinom | Adenokarzinom | 2 |
| NEO | Fibroneoplasie | Fibroneoplasie | 1 |
| NEO | Fibrosarkom | Fibrosarkom | 1 |
| NEO | Karzinom | Karzinom, Karzinoms | 2 |
| NEO | carcinoma | carcinoma | 1 |
| NEO | Colonkarzinom | Colonkarzinom | 1 |
| NEO | Lebermetastasen | Lebermetastasen | 1 |
| NEO | Lungenmetastasen | Lungenmetastasen | 4 |
| NEO | Lymphommetastase | Lymphommetastase | 1 |
| NEO | Lymphosarkom | Lymphosarkoms, Lymphosarkom | 27 |
| NEO | Metastasen | Metastasen, metastases | 10 |
| NEO | Metastasierung | Metastasierung | 1 |
| NEO | Neoplasie | Neoplasie, Neoplasien | 14 |
| NEO | neoplasia | neoplasia | 1 |
| NEO | neoplasm | neoplasm | 13 |
| NEO | Neoplasstic | Neoplasstic | 1 |
| NEO | neoplastic | neoplastischer, neoplastic, neoplastische, neoplastischen | 82 |
| NEO | neoplatische | neoplatische | 2 |
| NEO | Osteosarkom | Osteosarkom | 1 |
| NEO | Papillombildung | Papillombildung | 1 |
| NEO | Plattenepithelkarzinom | Plattenepithelkarzinom, Plattenepithelkarzinoms | 4 |
| NEO | Polyp | Polyp, Polypen | 7 |
| NEO | Sarkom | Sarkom | 1 |
| NEO | sarcoplasm | sarcoplasm | 1 |
| NEO | Tumor | Tumors, Tumor, tumor | 14 |
| NEO | Tumorgewebe | Tumorgewebe | 4 |
| NEO | Tumorknoten | Tumorknoten | 1 |
| NEO | Tumormetastasen | Tumormetastasen | 1 |
| NEO | Uterusadenokarzinomen | Uterusadenokarzinomen | 1 |
| NEO | Uteruskarzinom | Uteruskarzinom | 1 |
| NEURO | Enzephalitiden | Enzephalitiden | 1 |
| NEURO | Encephalitis | Encephalitis, encephalitis, enzephalitis, Enzephalitis | 52 |
| NEURO | Hirnstammenzephalitis | Hirnstammenzephalitis | 1 |
| NEURO | Hirntammencephalitis | Hirntammencephalitis | 1 |
| NEURO | Meningo-Encephalitis | Meningo-Encephalitis, Meningo-Enzephalitis | 10 |
| NEURO | Meningoencephalitis | Meningoenzephalitis, meningoencephalitis, Meningoencephalitis | 63 |
| NEURO | Meningoencephalomyelitis | Meningoenzephalomyelitis, Meningoencephalomyelitis, meningoencephalomyelitis | 12 |
| NEURO | Hydrocephalie | Hydrocephalie | 1 |
| NEURO | Hydrocephalus | Hydrozephalus, Hydrocephalus | 28 |
| NEURO | Wassermangel-Encephalitis | Wassermangel-Encephalitis | 1 |
| NEURO | Wassermangel-Encephalopathie | Wassermangel-Enzephalopathie, Wassermangel-Encephalopathie | 4 |
| NEURO | Meiningitis | Meiningitis | 1 |
| NEURO | Meningits | Meningits | 1 |
| NEURO | Meningitiden | Meningitiden | 2 |
| NEURO | Meningitis | Meningitis | 181 |
| NEURO | Meningo-Chorio-Ependymitis | Meningo-Chorio-Ependymitis | 4 |
| NEURO | Meningo-Encephalitis | Meningo-Encephalitis, Meningo-Enzephalitis | 10 |
| NEURO | Meningo-Enzephalo-Myelitis | Meningo-Enzephalo-Myelitis | 1 |
| NEURO | Meningo-Ependymitis | Meningo-Ependymitis | 3 |
| NEURO | Meningoencephalitis | Meningoenzephalitis, meningoencephalitis, Meningoencephalitis | 63 |
| NEURO | Meningoencephalomyelitis | Meningoenzephalomyelitis, Meningoencephalomyelitis, meningoencephalomyelitis | 12 |
| NEURO | Meningoependymitis | Meningoependymitis | 7 |
| NEURO | Meningomyelitis | meningomyelitis, Meningomyelitis | 5 |
| NEURO | Pachymeningitis | Pachymeningitis | 4 |
| NEURO | Myelitis | Myelitis | 5 |
| NEURO | Myelopathie | Myelopathie | 4 |
| NEURO | Myelopathy | myelopathy, Myelopathy | 2 |
| NEURO | Polio-Enzephalomyelitis | Polio-Enzephalomyelitis | 2 |
| NEURO | Polioencephalomyelitis | Polioencephalomyelitis | 4 |
| NEURO | Polyoencephalomyelitis | Polyoencephalomyelitis | 8 |
| NEURO | Angiopathie | Angiopathie | 17 |
| NEURO | Axonophagie | Axonophagie | 3 |
| NEURO | axonophagia | axonophagia | 1 |
| NEURO | Blasenlähmung | Blasenlähmung | 1 |
| NEURO | Chorioependymitis | Chorioependymitis | 1 |
| NEURO | Enzepalopathie | Enzepalopathie | 1 |
| NEURO | Encephalomalazie | Encephalomalazie, Enzephalomalazie, Enzephalomalazien | 6 |
| NEURO | Encephalopathie | Enzephalopathie, Encephalopathie | 9 |
| NEURO | Gehirnabszesse | Gehirnabszesse | 1 |
| NEURO | Hirnabszess | Hirnabszess | 3 |
| NEURO | Hyophysenabzess | Hyophysenabzess | 1 |
| NEURO | Hypophysenabszess | Hypophysenabszess | 9 |
| NEURO | Hypophysenabzess | Hypophysenabzess | 2 |
| NEURO | Lähmung | Lähmung | 3 |
| NEURO | Neuritis | Neuritis | 6 |
| NEURO | Neuropathie | Neuropathie | 1 |
| NEURO | Paralyse | Paralyse | 2 |
| NEURO | Poliomyelitis | Poliomyelitis, poliomyelitis | 4 |
| NEURO | Polymyelitis | Polymyelitis | 1 |
| NEURO | Polyomyelitis | Polyomyelitis | 1 |
| NEURO | Polyradiculitis | Polyradiculitis | 1 |
| NEURO | Polyradiculopathie | Polyradiculopathie | 1 |
| NEURO | Radiculo-Neuritis | Radiculo-Neuritis | 1 |
| NEURO | Speroids | Speroids | 1 |
| NEURO | Sphäroide | Sphäroide, Sphäroiden | 5 |
| NEURO | Spheroid | spheroids, Spheroide, Spheroiden, Spheroid | 17 |
| NEURO | Wallerian | Wallerian | 5 |
| NEURO | Wallerian-like | Wallerian-like | 1 |
| NEURO | Wallerian-type | Wallerian-type | 1 |
| NEURO | Wallersche | Wallersche | 5 |
| NEURO | Polio-Enzephalomyelitis | Polio-Enzephalomyelitis | 2 |
| NEURO | Polioencephalomyelitis | Polioencephalomyelitis | 4 |
| NEURO | Polyoencephalomyelitis | Polyoencephalomyelitis | 8 |
| NEURO | Gehirnödem | Gehirnödem | 2 |
| NEURO | Hirnödem | Hirnödem, Hirnoedem | 7 |
| NEURO | Hirnödems | Hirnödems | 2 |
| NEURO | Hydranencephalie | Hydranencephalie | 3 |
| NEURO | CCN | CCN | 7 |
| NEURO | Kortikozerebralnekrose | Kortikozerebralnekrose | 1 |
| NEURO | Polioencephalomalacie | Polioencephalomalacie, Polioencephalomalazie, Polioenzephalomalazie, Polioenzephalomalacie | 20 |
| NEURO | polioencephalomalacia | polioencephalomalacia | 1 |
| NEURO | Polioencephalomazie | Polioencephalomazie | 1 |
| NEURO | Poliomyelomalazie | Poliomyelomalazie, Poliomyelomalazien | 6 |
| NEURO | Polyencephalomalazie | Polyencephalomalazie, Polyenzephalomalazie | 2 |
| NEURO | Rückenmarkkompression | Rückenmarkkompression | 1 |
| NEURO | Rückenmarkskompression | Rückenmarkskompression | 10 |
| NEURO | Satellitose | Satellitose | 9 |
| NEURO | Sattelitose | Sattelitose | 2 |
| NEURO | Wassermangel-Encephalopathie | Wassermangel-Enzephalopathie, Wassermangel-Encephalopathie | 4 |
| NEURO | Cerebro-Cortical-Nekrose | Cerebro-Cortical-Nekrose | 1 |
| NEURO | Cerebrocorticalnekrose | Zerebrokortikalnekrose, Cerebrocorticalnekrose, Cerebrokortikalnekrose | 5 |
| OTHER | Blepharitis | Blepharitis | 1 |
| OTHER | Keratitis | keratitis, Keratitis | 17 |
| OTHER | Kerato-Conjunctivitis | Kerato-Conjunctivitis, Kerato-Konjunktivitis | 2 |
| OTHER | Keratokonjunktivitis | Keratokonjunktivitis | 3 |
| OTHER | Corneaödem | Corneaödem, Korneaödem | 2 |
| OTHER | Korneaperforation | Korneaperforation | 1 |
| OTHER | Uveitis | Uveitis, uveitis | 13 |
| OTHER | Epidermitis | Epidermitis | 21 |
| OTHER | Hautulcus | Hautulcus | 4 |
| OTHER | Hautulcera | Hautulzera, Hautulcera | 9 |
| OTHER | Hautulzeration | Hautulzerationen, Hautulzeration | 26 |
| OTHER | Erdrücken | Erdrücken | 78 |
| OTHER | erdrückt | erdrückt | 3 |
| OTHER | hidrothorax | hidrothorax | 1 |
| OTHER | Hydothorax | Hydothorax | 1 |
| OTHER | Hydrothorax | Hydrothorax | 116 |
| OTHER | Hydrothrax | Hydrothrax | 1 |
| OTHER | Hydrotorax | Hydrotorax | 1 |
| OTHER | Inanition | Inanition | 74 |
| OTHER | Inantion | Inantion | 1 |
| OTHER | -Vergiftung | -Vergiftung | 2 |
| OTHER | Adlerfarnvergiftung | Adlerfarnvergiftung, Adlerfarnvergiftungen | 3 |
| OTHER | Aflatoxin-Vergiftung | Aflatoxin-Vergiftung | 1 |
| OTHER | Ammoniak-Intoxikation | Ammoniak-Intoxikation | 1 |
| OTHER | Ammoniakvergiftung | Ammoniakvergiftung | 1 |
| OTHER | Arsenvergiftung | Arsenvergiftung | 1 |
| OTHER | Bleiintoxikation | Bleiintoxikationen, Bleiintoxikation | 2 |
| OTHER | Bleintoxikation | Bleintoxikation | 1 |
| OTHER | Bleivergiftung | Bleivergiftungen, Bleivergiftung | 7 |
| OTHER | D-Lactat-Intoxikation | D-Lactat-Intoxikation | 1 |
| OTHER | D-Vergiftung | D-Vergiftung | 2 |
| OTHER | Eichenvergiftung | Eichenvergiftungen, Eichenvergiftung | 2 |
| OTHER | Eisen-Intoxikation | Eisen-Intoxikation | 2 |
| OTHER | Eisenintoxikation | Eisenintoxikation | 4 |
| OTHER | Furazolidon-Vergiftung | Furazolidon-Vergiftung | 1 |
| OTHER | Güllengasvergiftung | Güllengasvergiftung | 1 |
| OTHER | Hahnenfussvergiftung | Hahnenfussvergiftung | 1 |
| OTHER | Harnstoff-Vergiftung | Harnstoff-Vergiftung | 2 |
| OTHER | Harnstoffvergiftung | Harnstoffvergiftung | 2 |
| OTHER | Intoxikation | Intoxikationen, Intoxikation, intoxication | 21 |
| OTHER | Jauchegasvergiftung | Jauchegasvergiftung | 7 |
| OTHER | Kochsalzvergiftung | Kochsalzvergiftung | 10 |
| OTHER | Kumarinvergiftung | Kumarinvergiftung | 1 |
| OTHER | Kupfervergiftung | Kupfervergiftung | 1 |
| OTHER | Leber-Intoxikation | Leber-Intoxikation | 1 |
| OTHER | Methaldehyde-Vergiftung | Methaldehyde-Vergiftung | 1 |
| OTHER | Mykotoxinevergiftung | Mykotoxinevergiftung | 1 |
| OTHER | Mykotoxinintoxikation | Mykotoxinintoxikation | 1 |
| OTHER | NaCl-Vergiftung | NaCl-Vergiftung | 1 |
| OTHER | Oleander | Oleanders, oleander, Oleander | 8 |
| OTHER | Oleander-Vergiftung | Oleander-Vergiftung | 1 |
| OTHER | Oleanderblätter | Oleanderblätter | 3 |
| OTHER | Pflanzenvergiftung | Pflanzenvergiftung | 3 |
| OTHER | Salzintoxikation | Salzintoxikation | 3 |
| OTHER | Salzvergiftung | Salzvergiftung | 2 |
| OTHER | Selen-Intoxikation | Selen-Intoxikation | 1 |
| OTHER | Selenintoxikation | Selenintoxikation | 2 |
| OTHER | Selenvergiftung | Selenvergiftung | 1 |
| OTHER | Süsskleevergiftung | Süsskleevergiftung | 1 |
| OTHER | Trichothecen-Intoxikation | Trichothecen-Intoxikation | 1 |
| OTHER | Vergiftung | Vergiftung | 20 |
| OTHER | Zottelwicken-Intoxikation | Zottelwicken-Intoxikation | 1 |
| OTHER | Coli-Mastitis | Coli-Mastitis | 1 |
| OTHER | masititis | masititis | 1 |
| OTHER | Mastiltis | Mastiltis | 1 |
| OTHER | Mastitis | Mastitis, mastitis | 92 |
| OTHER | Pansentrinken | Pansentrinker, Pansentrinkens, Pansentrinken | 32 |
| OTHER | Pansentrinker-Kalb | Pansentrinker-Kalb | 1 |
| OTHER | Pansentrinker-Syndrom | Pansentrinker-Syndrom | 2 |
| OTHER | Pansentrinkerkalb | Pansentrinkerkalb | 2 |
| OTHER | -urachitis | -urachitis | 1 |
| OTHER | Omphalitis | Omphalitis | 43 |
| OTHER | Omphalo-Urachitis | Omphalo-Urachitis | 1 |
| OTHER | Omphaloarteriitis | Omphaloarteriitis | 3 |
| OTHER | Omphaloarteritis | Omphaloarteritis | 18 |
| OTHER | omphalophlebiltis | omphalophlebiltis | 1 |
| OTHER | Omphalophlebitis | omphalophlebitis, Omphalophlebitis | 84 |
| OTHER | Omphalourachitis | Omphalourachitis | 5 |
| OTHER | umphalophlebitis | umphalophlebitis | 1 |
| OTHER | Urachitis | Urachitis | 13 |
| OTHER | Otitis | Otitis | 35 |
| OTHER | Hautperforation | Hautperforation | 6 |
| OTHER | -Sepsis | -Sepsis | 1 |
| OTHER | Bordetellensepsis | Bordetellensepsis | 1 |
| OTHER | enterotoxämischen | enterotoxaemischen, enterotoxämischen | 2 |
| OTHER | Enterotoxämie | Enterotoxaemie, Enterotoxämie | 17 |
| OTHER | Haemophilus-parasuis-Sepsis | Haemophilus-parasuis-Sepsis | 1 |
| OTHER | hyicus-Sepsis | hyicus-Sepsis | 1 |
| OTHER | Campylobacter-Sepsis | Campylobacter-Sepsis | 1 |
| OTHER | Klebsiellen-Sepsis | Klebsiellen-Sepsis | 1 |
| OTHER | Coli-Sepsis | Coli-Sepsis, coli-Sepsis | 101 |
| OTHER | Coli-Septikämie | Coli-Septikämie, Koli-Septikämie, coli-Septikämie | 9 |
| OTHER | Coli-Septikemie | Coli-Septikemie | 1 |
| OTHER | Colisepsis | Colisepsis | 2 |
| OTHER | Coliseptikämie | Coliseptikämie | 5 |
| OTHER | parasuis-Sepsis | parasuis-Sepsis | 10 |
| OTHER | Pasteurellen-Sepsis | Pasteurellen-Sepsis | 3 |
| OTHER | Rotlauf-Sepsis | Rotlauf-Sepsis | 8 |
| OTHER | Rotlaufsepsis | Rotlaufsepsis | 1 |
| OTHER | Sepikämie | Sepikämie | 1 |
| OTHER | Sepitkämie | Sepitkämie | 1 |
| OTHER | Sepsis | sepsis, Sepsis | 267 |
| OTHER | septecemia | septecemia | 1 |
| OTHER | septikämisch | septikämischer, Septikämische, septikämische, septikämisch, septikämischen, septikämisches | 19 |
| OTHER | Septikämie | Septikämie, Septikämien | 183 |
| OTHER | Septikämiekomponente | Septikämiekomponente | 1 |
| OTHER | septikämisch-embolische | septikämisch-embolische | 1 |
| OTHER | septicémie | septicémie | 1 |
| OTHER | Septicaemie | Septicaemie | 1 |
| OTHER | Septicemia | septicemia, Septicemia | 4 |
| OTHER | Strepptokken-Sepsis | Strepptokken-Sepsis | 1 |
| OTHER | Streptokokken-Sepsis | Streptokokken-Sepsis | 80 |
| OTHER | Streptokokken-Septikämie | Streptokokken-Septikämie | 2 |
| OTHER | Streptokokkensepsis | Streptokokkensepsis | 3 |
| OTHER | Streptococcus-Sepsis | Streptococcus-Sepsis | 1 |
| OTHER | Streptococcen-Sepsis | Streptococcen-Sepsis | 2 |
| OTHER | suis-Sepsis | suis-Sepsis | 2 |
| OTHER | suis-Septikämie | suis-Septikämie | 1 |
| OTHER | Dematitis | Dematitis | 1 |
| OTHER | Dermatistis | Dermatistis | 1 |
| OTHER | Dermatitis | Dermatitis, dermatitis | 162 |
| REPRO | Abort | Aborts, Abort, Abortes, Aborte, Aborten | 303 |
| REPRO | Abort-Ursache | Abort-Ursache, Abort-Ursachen | 9 |
| REPRO | Aborterreger | Aborterregern, Aborterreger, aborterreger | 89 |
| REPRO | Aborterrreger | Aborterrreger | 1 |
| REPRO | Abortgeschehen | Abortgeschehen | 11 |
| REPRO | abortiert | abortiertes, abortierten, abortierte, Abortierte, abortiert, abortierter, Abortierter | 48 |
| REPRO | Abortion | Abortion, abortion | 5 |
| REPRO | Abortsursache | Abortsursache | 2 |
| REPRO | Abortuntersuche | Abortuntersuchung, Abortuntersuche, Abortuntersuchungen | 11 |
| REPRO | Abortursache | Abortursache, Abortursachen | 171 |
| REPRO | Abortus | Abortus, abortus | 113 |
| REPRO | Abortusursache | Abortusursache | 7 |
| REPRO | Brucella-Abort | Brucella-Abort | 1 |
| REPRO | BVD-Abort | BVD-Abort | 1 |
| REPRO | Chlamydienabort | Chlamydienabort | 1 |
| REPRO | Frühabort | Frühabort, Frühaborten | 2 |
| REPRO | caninum-Abort | caninum-Abort | 1 |
| REPRO | Coxiellen-Abort | Coxiellen-Abort | 1 |
| REPRO | Mumie | Mumie | 1 |
| REPRO | Mumifikation | Mumifikation | 29 |
| REPRO | Mummifikation | Mummifikation | 11 |
| REPRO | Neospoa-PCR | Neospoa-PCR | 1 |
| REPRO | Neospora-Abort | Neospora-Abort | 6 |
| REPRO | Neospora-PCR | Neospora-PCR | 73 |
| REPRO | Neosporose | Neosporose | 25 |
| REPRO | Neospra-PCR | Neospra-PCR | 1 |
| REPRO | Placentitis | Placentitis, placentitis, Plazentitis | 165 |
| REPRO | pyogenes-Abort | pyogenes-Abort | 1 |
| REPRO | Rinderabort | Rinderabortes, Rinderabort | 7 |
| REPRO | Rinderfötusabort | Rinderfötusabort | 1 |
| REPRO | Schweineabort | Schweineabort | 2 |
| REPRO | Spätabort | Spätabort | 1 |
| REPRO | Streptokokken-Abort | Streptokokken-Abort | 2 |
| REPRO | totgeborene | totgeborenes, Totgeborenes, totgeborene, totgeborener | 18 |
| REPRO | Totgeburt | Totgeburten, Totgeburt | 34 |
| REPRO | Toxoplasma-Abort | Toxoplasma-Abort | 1 |
| REPRO | Zwillingsabort | Zwillingsabort | 1 |
| REPRO | Distokie | Distokie | 1 |
| REPRO | Dystokie | Dystokie | 8 |
| REPRO | Endometritis | endometritis, Endometritis | 78 |
| REPRO | Metritis | Metritis | 13 |
| REPRO | Perimetritis | perimetritis, Perimetritis | 3 |
| REPRO | Nachgeburtsverhaltung | Nachgeburtsverhaltung | 2 |
| REPRO | Plazentaretention | Plazentaretention | 5 |
| REPRO | Hyperestrogenismus | Hyperestrogenismus | 1 |
| REPRO | Hyperöstrogenismus | Hyperoestrogenismus, Hyperöstrogenismus | 7 |
| REPRO | Ovarialzysten | Ovarialzysten | 1 |
| REPRO | testicular lesions | testicular lesion | 1 |
| REPRO | Uterusperforation | Uterusperforation | 3 |
| REPRO | Uteruswandperforation | Uteruswandperforation | 1 |
| REPRO | Torsio uteri | Torsio uteri | 4 |
| REPRO | Torsion der Uterushörner | Torsion der Uterushörner | 1 |
| REPRO | Uterustorsion | Uterustorsion | 4 |
| REPRO | Uterusperforation | Uterusperforation | 3 |
| REPRO | Uteruswandperforation | Uteruswandperforation | 1 |
| REPRO | Perivaginitis | Perivaginitis | 2 |
| REPRO | Vaginitis | Vaginitis | 7 |
| REPRO | Vulvo-vaginitis | Vulvo-vaginitis | 1 |
| RESPI | Bonchitis | Bonchitis | 1 |
| RESPI | Bronchitis | Bronchitis, bronchitis | 713 |
| RESPI | Bronchitits | Bronchitits | 1 |
| RESPI | EitrigeBronchitis | EitrigeBronchitis | 1 |
| RESPI | Peribronchitis | Peribronchitis | 114 |
| RESPI | Bronchiolits | Bronchiolits | 1 |
| RESPI | Bronchiolitis | Bronchiolitis, bronchiolitis | 177 |
| RESPI | Peribronchiolitis | Peribronchiolitis | 117 |
| RESPI | Aspiration-Bronchopneumonie | Aspiration-Bronchopneumonie | 1 |
| RESPI | Bochiopneumonie | Bochiopneumonie | 1 |
| RESPI | Bonchopneumonie | Bonchopneumonie | 1 |
| RESPI | Brochiopneumonia | Brochiopneumonia | 1 |
| RESPI | Brochniopneumonie | Brochniopneumonie | 1 |
| RESPI | Brochnopneumonie | Brochnopneumonie | 2 |
| RESPI | Brochopneumonie | Brochopneumonie | 5 |
| RESPI | brochopneumonia | brochopneumonia | 1 |
| RESPI | Bronchiopneumonie | Bronchiopneumonie | 2 |
| RESPI | Broncho-Pneumonie | Broncho-Pneumonie | 4 |
| RESPI | Bronchoneumonie | Bronchoneumonie | 2 |
| RESPI | bronchoneumonia | bronchoneumonia | 1 |
| RESPI | Bronchopenumonie | Bronchopenumonie | 2 |
| RESPI | Bronchopnemonie | Bronchopnemonie | 1 |
| RESPI | Bronchopneumomie | Bronchopneumomie | 1 |
| RESPI | bronchopneumonische | bronchopneumonische | 1 |
| RESPI | Bronchopneumonie | Bronchopneumonie, bronchopneumonie | 1322 |
| RESPI | Bronchopneumonia | Bronchopneumonia, bronchopneumonia | 24 |
| RESPI | Bronchopneuonie | Bronchopneuonie | 1 |
| RESPI | Bronchopnuemonie | Bronchopnuemonie | 1 |
| RESPI | bronchpneumonia | bronchpneumonia | 1 |
| RESPI | Pleurobronchonpneumonie | Pleurobronchonpneumonie | 1 |
| RESPI | Pleurobronchopneumonie | Pleurobronchopneumonie | 2 |
| RESPI | suis-Bronchopneumonie | suis-Bronchopneumonie | 1 |
| RESPI | Larnygitis | Larnygitis | 1 |
| RESPI | Laryngitis | Laryngitis | 20 |
| RESPI | Actinobacillus-Pleuropneumonie | Actinobacillus-Pleuropneumonie | 15 |
| RESPI | Aspirationpneumonie | Aspirationpneumonie | 1 |
| RESPI | Aspirations-Pneumonie | Aspirations-Pneumonie | 1 |
| RESPI | Aspirationspeumonie | Aspirationspeumonie | 1 |
| RESPI | Aspirationspneumonie | Aspirationspneumonie | 63 |
| RESPI | Bordetella-Pneumonie | Bordetella-Pneumonie | 2 |
| RESPI | Bordetellen-Pneumonie | Bordetellen-Pneumonie | 86 |
| RESPI | Bordetellenpneumonie | Bordetellenpneumonie | 2 |
| RESPI | Bordetellose | Bordetellose | 8 |
| RESPI | Bordotellen-Pneumonie | Bordotellen-Pneumonie | 1 |
| RESPI | Futter-Aspiration | Futter-Aspiration | 1 |
| RESPI | Futteraspiration | Futteraspiration | 9 |
| RESPI | Herdpneumonie | Herdpneumonie | 8 |
| RESPI | Penumonie | Penumonie | 2 |
| RESPI | Peumonie | Peumonie | 1 |
| RESPI | Pneumomie | Pneumomie | 1 |
| RESPI | Pneumonie | Pneumonie, Pneumonien, pneumonie | 1202 |
| RESPI | pneumonia | pneumoniae, Pneumonia, pneumonia | 38 |
| RESPI | pneumonias | pneumonias | 1 |
| RESPI | Pneumonitis | Pneumonitis | 1 |
| RESPI | Wurmpneumonie | Wurmpneumonie | 2 |
| RESPI | Pleuropneumonie | Pleuropneumonie, Pleuropneumonien, pleuropneumonie | 43 |
| RESPI | Retropharyngitis | Retropharyngitis | 2 |
| RESPI | Einschlusskörperchen-Rhinitis | Einschlusskörperchen-Rhinitis | 9 |
| RESPI | Einschlusskörperchenrhinitis | Einschlusskörperchenrhinitis | 2 |
| RESPI | athophicans | athophicans | 1 |
| RESPI | athrophicans | athrophicans, athrophikans | 2 |
| RESPI | Atrophicans | atrophicans, Atrophicans | 148 |
| RESPI | Rhinits | Rhinits | 1 |
| RESPI | Rhinitis | Rhinitis | 234 |
| RESPI | Rhintis | Rhintis | 1 |
| RESPI | Sinusits | Sinusits | 1 |
| RESPI | Sinusiti | Sinusiti | 1 |
| RESPI | Sinusitis | Sinusitis | 8 |
| RESPI | Tracheitis | Tracheitis, tracheitis | 26 |
| RESPI | Laryngo-Tracheitis | Laryngo-Tracheitis | 5 |
| RESPI | Laryngotracheitis | Laryngotracheitis | 4 |
| SEROSA | Epikardis | Epikardis | 1 |
| SEROSA | Epicarditis | Epikarditis, epicarditis, Epicarditis | 70 |
| SEROSA | Epikard-Perikarditis | Epikard-Perikarditis | 1 |
| SEROSA | Nischenpericarditis | Nischenpericarditis | 1 |
| SEROSA | Perikardfibrose | Perikardfibrose | 1 |
| SEROSA | Pericarditis | Pericarditis, pericarditis, Perikarditis | 487 |
| SEROSA | Perikarditits | Perikarditits | 1 |
| SEROSA | Perkarditis | Perkarditis | 2 |
| SEROSA | Pleuro-Perikarditis | Pleuro-Perikarditis | 1 |
| SEROSA | Pleuroperikarditis | Pleuroperikarditis | 41 |
| SEROSA | Pleurpericarditis | Pleurpericarditis | 1 |
| SEROSA | Peritonits | peritonits, Peritonits | 3 |
| SEROSA | Peritonitis | Peritonitis, peritonitis | 1210 |
| SEROSA | Peritonitits | Peritonitits | 1 |
| SEROSA | Pertionitis | Pertionitis | 1 |
| SEROSA | Pertonitis | Pertonitis | 1 |
| SEROSA | Reticulo-Peritonitis | reticulo-peritonitis, Reticulo-Peritonitis, Retikulo-Peritonitis | 12 |
| SEROSA | Retikulo-Peritonitis-traumatica | Retikulo-Peritonitis-traumatica | 1 |
| SEROSA | Reticuloperitonitis | reticuloperitonitis, Retikuloperitonitis, Reticuloperitonitis | 71 |
| SEROSA | Retiukuloperitonitis | Retiukuloperitonitis | 1 |
| SEROSA | Retroperitonitis | retroperitonitis, Retroperitonitis | 4 |
| SEROSA | Pleuritis | Pleuritis, pleuritis | 736 |
| SEROSA | Pleuro-Perikarditis | Pleuro-Perikarditis | 1 |
| SEROSA | Pleuroperikarditis | Pleuroperikarditis | 41 |
| SEROSA | Pleuropneumonie | Pleuropneumonie, Pleuropneumonien, pleuropneumonie | 43 |
| SEROSA | Pleurpericarditis | Pleurpericarditis | 1 |
| SEROSA | Pleurtis | Pleurtis | 1 |
| SEROSA | Pleurtitis | Pleurtitis | 1 |
| SEROSA | Poliarthritis | Poliarthritis | 1 |
| SEROSA | Polyarhtritis | Polyarhtritis | 1 |
| SEROSA | Polyarthrits | Polyarthrits | 1 |
| SEROSA | Polyarthritis | polyarthritis, Polyarthritis | 407 |
| SEROSA | Polyartritis | Polyartritis | 2 |
| SEROSA | Polyathritis | Polyathritis | 1 |
| SEROSA | Poyarthritis | Poyarthritis | 1 |
| SEROSA | Poylarthritis | Poylarthritis | 1 |
| SEROSA | Rotlauf-Polyarthritis | Rotlauf-Polyarthritis | 5 |
| SEROSA | Staphylokokken-Polyarthritis | Staphylokokken-Polyarthritis | 1 |
| SEROSA | Plyserositis | Plyserositis | 1 |
| SEROSA | Polyserosits | Polyserosits | 2 |
| SEROSA | Polyserositiden | Polyserositiden | 1 |
| SEROSA | Polyserositis | Polyserositis, polyserositis | 349 |
| SEROSA | Polyserositis-Problemen | Polyserositis-Problemen | 1 |
| SEROSA | Polyserositiserreger | Polyserositiserreger | 1 |
| SEROSA | Polyserositisproblem | Polyserositisproblem | 1 |
| SEROSA | Poyserositis | Poyserositis | 1 |
| SEROSA | Serositis | Serositis, serositis | 25 |
| SEROSA | Streptokokken-Polyserositis | Streptokokken-Polyserositis | 1 |
| URI | Pyelozystitis | Pyelozystitis | 12 |
| URI | Cystitis | Cystitis, Zystitis | 50 |
| URI | Hydronephrose | Hydronephrose | 35 |
| URI | Hydronephrosis | Hydronephrosis | 1 |
| URI | Ureterektopie | Ureterektopie | 3 |
| URI | Nierenfibrose | Nierenfibrose | 2 |
| URI | Nierenzyste | Nierenzyste, Nierenzysten | 74 |
| URI | Nierenzytse | Nierenzytse | 1 |
| URI | Schrumpfnieren | Schrumpfnieren | 1 |
| URI | Tubulidegeneration | Tubulidegeneration | 4 |
| URI | Tubulinekrose | Tubulinekrose | 1 |
| URI | Tubulodegeneration | Tubulodegeneration | 3 |
| URI | Tubulonekrose | Tubulonekrose | 2 |
| URI | Tubulonephrose | Tubulonephrose | 19 |
| URI | Tubulusdegeneration | Tubulusdegeneration | 13 |
| URI | Tubulusnekrose | Tubulusnekrose | 4 |
| URI | Perinephritis | Perinephritis | 3 |
| URI | Pyelitis | Pyelitis | 7 |
| URI | Pyelonephritis | pyelonephritis, Pyelonephritis | 31 |
| URI | Pyonephrose | Pyonephrose | 4 |
| URI | Tubulonephritis | tubulonephritis, Tubulonephritis | 6 |
| URI | Harnphlegmone | Harnphlegmone | 2 |
| URI | Megaureter | Megaureter | 1 |
| URI | urethritis | Urethritis, urethritis | 7 |
| URI | Blasenruptur | Blasenruptur | 2 |
| URI | Harnblasenruptur | Harnblasenruptur | 7 |
| URI | Urethra-Ruptur | Urethra-Ruptur | 1 |
| URI | Harnkonkremente | Harnkonkremente, Harnkonkrementen | 3 |
| URI | Nephrolithe | Nephrolithe | 2 |
| URI | Nephrolithiasis | Nephrolithiasis | 2 |
| URI | Nierenstein | Nierenstein, Nierensteine | 8 |
| URI | Urat-Kristallen | Urat-Kristallen | 1 |
| URI | Urat-Kristall-Ablagerung | Urat-Kristall-Ablagerung | 1 |
| URI | Uratkristalle | Uratkristallen, Uratkristalle | 51 |
| URI | Uratkristall-Ablagerungen | Uratkristall-Ablagerungen | 1 |
| URI | Uratkristallablagerung | Uratkristallablagerung, Uratkristallablagerungen | 5 |
| URI | Urinkristalle | Urinkristalle | 2 |
| URI | Urolith | Urolithen, Urolithe, Urolith | 6 |
| URI | Urolithiase | Urolithiase | 2 |
| URI | Urolithiasis | Urolithiasis | 12 |
| URI | Glomerulo-Nephritis | Glomerulo-Nephritis | 1 |
| URI | Glomerulonephritis | Glomerulonephritis | 142 |
| URI | Glomerulonephritits | Glomerulonephritits | 1 |
| URI | Glumerulonephritis | Glumerulonephritis | 1 |
| URI | Nephritiden | Nephritiden | 1 |
| URI | Nephritis | nephritis, Nephritis | 356 |
| URI | Nerphritis | Nerphritis | 2 |
